# Managing toxicities associated with immune checkpoint inhibitors: consensus recommendations from the Society for Immunotherapy of Cancer (SITC) Toxicity Management Working Group

**DOI:** 10.1186/s40425-017-0300-z

**Published:** 2017-11-21

**Authors:** I. Puzanov, A. Diab, K. Abdallah, C. O. Bingham, C. Brogdon, R. Dadu, L. Hamad, S. Kim, M. E. Lacouture, N. R. LeBoeuf, D. Lenihan, C. Onofrei, V. Shannon, R. Sharma, A. W. Silk, D. Skondra, M. E. Suarez-Almazor, Y. Wang, K. Wiley, H. L. Kaufman, M. S. Ernstoff, on behalf of the Society for Immunotherapy of Cancer Toxicity Management Working Group, Jeff Anderson, Deborah Arrindell, Stephanie Andrews, Joan Ballesteros, Janie Boyer, Daniel Chen, David Chonzi, Ion Cotarla, Renato Cunha, Marianne Davies, Michelle Dawson, Adam Dicker, Lisa Eifler, Andrew Ferguson, Cristiano Ferlini, Stanley Frankel, William Go, Celestine Gochett, Jenna Goldberg, Priscila Goncalves, Trishna Goswami, Nancy Gregory, James L. Gulley, Vinny Hayreh, Nicole Helie, William Holmes, Jer-Yuan Hsu, Ramy Ibrahim, Cecilia Larocca, Kimberly Lehman, Sergio Ley-Acosta, Olivier Lambotte, Jason Luke, Joan McClure, Elisabete Michelon, Mary Nakamura, Kiran Patel, Bilal Piperdi, Zeshaan Rasheed, Dan Reshef, Joanne Riemer, Caroline Robert, Makan Sarkeshik, Ann Saylors, Judy Schreiber, Kim Shafer-Weaver, William Sharfman, Elad Sharon, Richard Sherry, Cyndy Simonson, Cherry Thomas, John A. Thompson, Elizabeth Trehu, Dina Tresnan, Michelle Turner, Darshan Wariabharaj, Ian Waxman, Lauren Wood, Lin Zhang, Pan Zheng

**Affiliations:** 1 Roswell Park Cancer Institute, Elm & Carlton Streets, Buffalo, NY 14263 USA; 2 University of Texas MD Anderson Cancer Center, Houston, TX USA; 3 Merck & Co., Inc., Upper Gwynedd, PA USA; 4 Johns Hopkins University, Baltimore, MD USA; 5 Bristol-Myers Squibb Company, New York, NY USA; 6 Memorial Sloan Kettering Cancer Center, New York, NY USA; 7 Dana Farber/Brigham and Women’s Cancer Center, Boston, MA USA; 8 Washington University in St Louis, St Louis, MO USA; 9 Indiana University, Indianapolis, IN USA; 10 University of Chicago, Chicago, IL USA; 11 Oncology Nursing Society, Pittsburgh, PA USA; 12 Massachusetts General Hospital, Boston, MA USA

**Keywords:** Immune-related adverse events, Toxicity, Immune checkpoint inhibitor

## Abstract

Cancer immunotherapy has transformed the treatment of cancer. However, increasing use of immune-based therapies, including the widely used class of agents known as immune checkpoint inhibitors, has exposed a discrete group of immune-related adverse events (irAEs). Many of these are driven by the same immunologic mechanisms responsible for the drugs’ therapeutic effects, namely blockade of inhibitory mechanisms that suppress the immune system and protect body tissues from an unconstrained acute or chronic immune response. Skin, gut, endocrine, lung and musculoskeletal irAEs are relatively common, whereas cardiovascular, hematologic, renal, neurologic and ophthalmologic irAEs occur much less frequently. The majority of irAEs are mild to moderate in severity; however, serious and occasionally life-threatening irAEs are reported in the literature, and treatment-related deaths occur in up to 2% of patients, varying by ICI. Immunotherapy-related irAEs typically have a delayed onset and prolonged duration compared to adverse events from chemotherapy, and effective management depends on early recognition and prompt intervention with immune suppression and/or immunomodulatory strategies. There is an urgent need for multidisciplinary guidance reflecting broad-based perspectives on how to recognize, report and manage organ-specific toxicities until evidence-based data are available to inform clinical decision-making. The Society for Immunotherapy of Cancer (SITC) established a multidisciplinary Toxicity Management Working Group, which met for a full-day workshop to develop recommendations to standardize management of irAEs. Here we present their consensus recommendations on managing toxicities associated with immune checkpoint inhibitor therapy.

**Electronic supplementary material:**

The online version of this article (10.1186/s40425-017-0300-z) contains supplementary material, which is available to authorized users.

## Background

Cancer immunotherapy has revolutionized the treatment of cancer [1, 2]. Currently, the most widely used approach is the administration of targeted monoclonal antibodies (mAbs) directed against regulatory immune checkpoint molecules that inhibit T cell activation [1]. At present, six immune checkpoint inhibitors (ICIs) are approved by the U.S Food and Drug Administration (FDA) for use in a variety of solid tumors, and one hematological malignancy (Hodgkin lymphoma) [3]. Ipilimumab, a fully human IgG1 mAb that blocks the cytotoxic T lymphocyte-antigen-4 (CTLA-4), a checkpoint inhibitor of T cell activation, was the first ICI approved, in 2011, for use in advanced melanoma [4]. Pembrolizumab and nivolumab, both engineered IgG4 mAbs that regulate T cell activation by blocking the protein programmed death 1 (PD-1), received FDA approval in patients with advanced melanoma in 2014 [5, 6] and the indications for both have subsequently expanded considerably. Indeed, in a landmark regulatory step, the FDA recently approved both pembrolizumab and nivolumab for use in certain patients with mismatch repair deficient (dMMR) and microsatellite instability high (MSI-H) cancers that have progressed following treatment with chemotherapy – the first such ‘tissue-agnostic’, biomarker-driven approvals granted [5, 6]. Both anti-PD-1 agents are associated with negligible antibody-dependent cell-mediated cytotoxicity (ADCC), a process that could be detrimental to the activation of T effector cells. After approval of nivolumab for the treatment of non-small cell lung carcinoma (NSCLC) in 2015, the first immunotherapy combination of ipilimumab plus nivolumab was granted approval later in 2015, again in advanced melanoma [5]. More recently, the FDA approved three new ICIs, atezolizumab, durvalumab and avelumab, all of which are antibodies directed against the protein programmed death-ligand 1 (PD-L1). Both atezolizumab and durvalumab are engineered IgG1 mAbs that include Fc modifications that eliminate ADCC, while avelumab includes a wildtype IgG1 framework with intact ADCC. Since May 2016, atezolizumab and durvalumab have both been approved for the treatment of NSCLC and urothelial carcinoma, and avelumab was approved for use in Merkel cell carcinoma and urothelial carcinoma [7–9].

Immune-related adverse events (irAEs) are discrete toxicities caused by non-specific activation of the immune system, and can affect almost any organ system. In some studies, the reported incidence is as high as 90% for any-grade irAEs due to single-agent ICI therapy [10], but meta-analysis indicates an overall incidence <75% with anti-CTLA-4 monotherapy (ipilimumab) [11], and ≤30% in phase 3 trials of anti-PD-1/PD-L1 agents [12–14]. IrAEs ≥ grade 3 severity occur in up to 43% of patients taking ipilimumab [10] and ≤20% taking PD-1/PD-L1 agents [12, 15]. The incidence of irAEs with ipilimumab and pembrolizumab is dose-dependent, with greater toxicity at higher dose levels; toxicity also varies between the adjuvant and metastatic disease settings [10, 16–19]. There is significant variance in definitions of toxicity severity across disciplines, as well as variation in how symptoms and signs that may be attributable to the same underlying pathophysiology are reported. This causes considerable difficulty in obtaining accurate data on incidence and prevalence based on clinical trials [12]. Nonetheless, the incidence of most irAEs with ICI monotherapy appears to be broadly similar across tumor types [12]. Some of the mechanisms that underpin the development of inflammatory toxicity – in particular those driven by CD8 T cell activity – overlap with those responsible for the drugs’ therapeutic effects. However, the exact pathogenesis of immune toxicity is not clear, and many other inflammatory cells, such as Th17 and other types of cells, are reported to be involved. The mechanism of toxicity may also vary by ICI, and may ultimately affect acuity, chronicity and management. In some cases, irAEs may occur in patients with durable responses to treatment; this association has not been fully ascertained [20, 21].

**Fig. 1:**
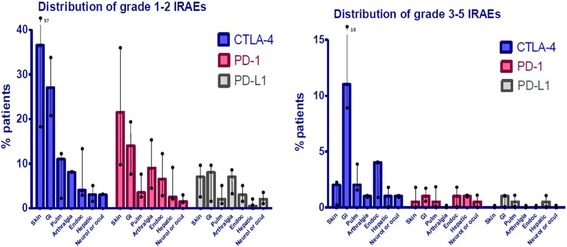
Distribution of mild and severe immune-related adverse events (irAEs) associated with immune checkpoint inhibitor therapy. [Adapted from [88]]

With increasing patient exposure to immunotherapy, the nature and range of irAEs is becoming more clearly defined, and several new but serious adverse events have been reported [22]. Skin, gut, endocrine, lung and musculoskeletal irAEs are relatively common, whereas, cardiovascular, hematologic, renal, neurologic and ophthalmologic irAEs are well-recognized but occur much less frequently (Fig. 1). Although the majority of irAEs are mild to moderate in severity, serious, occasionally life-threatening irAEs (e.g., severe colitis, pneumonitis, encephalitis, toxic epidermal necrolysis, myocarditis, and autoimmune type I diabetes mellitus [T1DM] presenting as diabetic ketoacidosis), are reported in the literature, and treatment-related deaths have been reported in up to 2% of patients in clinical trials [14, 23, 24]. As life-threatening irAEs are rare, and may mimic other better-known conditions, there is growing recognition of the need to educate both the oncology and general medical communities in recognizing and instituting urgent and appropriate treatment of these conditions.

**Fig. 2:**
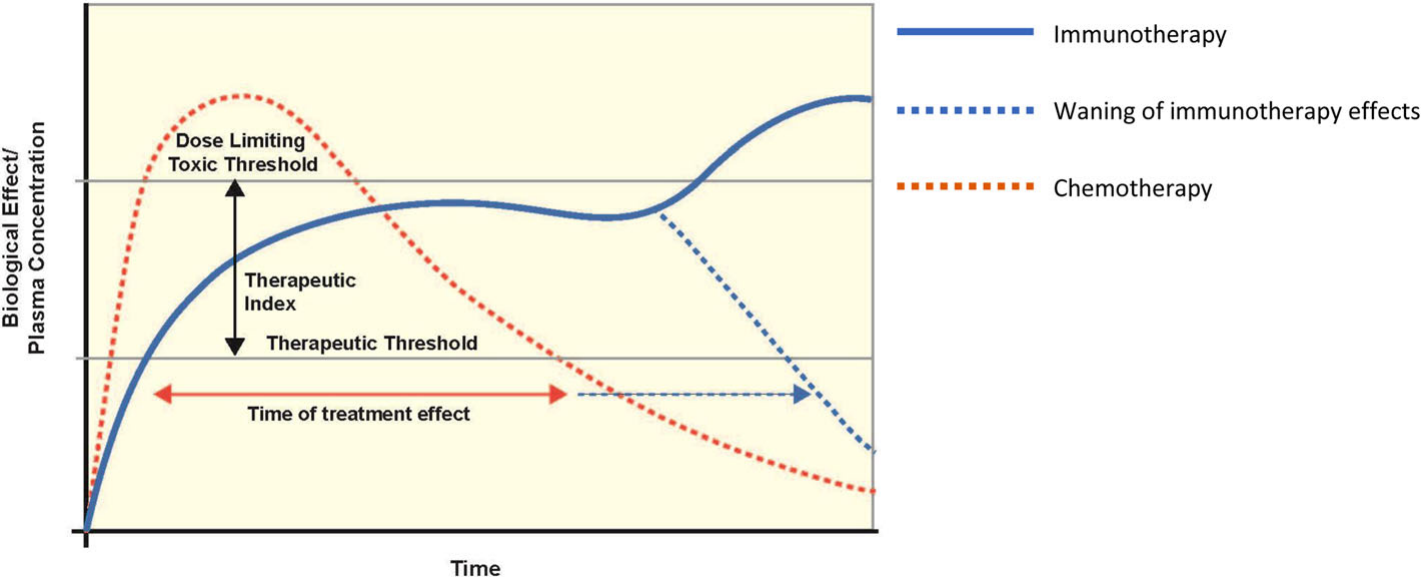
Pharmacokinetic/pharmacodynamic differences between chemotherapy and immunotherapy. Reproduced with permission from [25]. Dotted blue line represents waning of the biological effects of immunotherapy over time, and solid blue line represents early or late toxic effects. Horizontal dotted blue arrow therefore represents duration of immunotherapy treatment benefit

Immune-related AEs resulting from immunotherapy can have a delayed onset and prolonged duration compared to adverse events resulting from chemotherapy (Fig. 2), in part due to pharmacodynamic differences. Moreover, the relationship between irAEs and dose/exposure remains to be fully established [25]. As such, clinicians must remain vigilant to the diverse clinical presentations of irAEs and the possibility that patients may present with irAEs late in the course of treatment, and – in some cases – months or even years after treatment discontinuation [26, 27]. Nonetheless, since diagnostic tests may be invasive and potentially costly, investigations should be undertaken judiciously and reserved for situations when the results will guide patient management. Table 1 provides a list of recommended tests to consider in all patients prior to initiating checkpoint inhibitor therapy.

**Table 1:** Pre-treatment evaluation and diagnostic tests to consider in all patients prior to initiating checkpoint inhibitor therapy

Routine pre-treatment screening
History ♦ Detailed questioning for autoimmune, infectious disease, endocrine and organ-specific disease history ♦ History of base line bowel habit (frequency of bowel movements, usual stool consistency) Blood tests ♦ CBC ♦ CMP ♦ TSH ♦ HbA1c ♦ Free T4 ♦ Total CK ♦ Infectious disease screen: HBsAg, HBsAb, HBcAb, hCAb, CMV antibody, T-spot test, HIV antibody, HIV antigen (p24)^a^ ♦ Fasting lipid profile Dermatologic examination ♦ Full skin and mucosal exam, taking note of the extent and type of lesions present Pulmonary tests ♦ Baseline oxygen saturation on room air and during ambulation Cardiac tests ♦ ECG ♦ Troponin I or T: baseline and weekly for 6 weeks^b^
Additional screening tests recommended in patients with pre-existing organ disease/at risk of organ-specific toxicity
Endocrine tests ♦ 8 am cortisol ♦ 8 am ACTH Cardiac tests ♦ Brain natriuretic peptide (BNP) or N-terminal pro B-type natriuretic peptide (NT pro-BNP) Pulmonary tests ♦ PFTs^c^ ♦ 6MWT^c^

In certain settings, some of these tests may not be readily available. Until their use is firmly supported by evidence, individual physician judgment is recommended

^a^These tests become very relevant if patients develop irAEs and require immunosuppressive treatment such as steroids and/or anti-TNFα treatment

^b^Given the rarity of cardiac toxicity, this may not be cost-effective as a routine test. . Baseline troponin should be measured although the follow up interval for re-testing is not determined. Any suspicious cardiopulmonary symptoms warrant repeat troponin and natriuretic testing in this population

^c^Given the rarity of pulmonary toxicity, pre-treatment PFTs and 6MWTs should considered in patients with pre-existing lung disease (chronic obstructive pulmonary disease, interstitial lung disease, sarcoidosis, pulmonary fibrosis etc.) and may not be feasible in all patients

ACTH, Adrenocorticotropic hormone; CBC, Complete blood count; CMP, Complete metabolic panel; CMV, Cytomegalovirus; CK, Creatine kinase; ECG, Electrocardiogram; HbA1c, Glycosylated hemoglobin; HBsAg, Hepatitis B surface antigen; HBsAb, Hepatitis B surface antibody; HBcAb, Hepatitis B core antibody; HCAb, Hepatitis C antibody; HIV, Human Immunodeficiency Virus; PFTs, Pulmonary function tests; TSH, Thyroid-stimulating hormone; T4, Thyroxine; 6MWT, 6 min walk test

Effective management of irAEs depends on early recognition and prompt intervention with immune suppression and/or immunomodulatory strategies appropriate to the affected organ and the severity of toxicity. Specialist physicians, nurses and pharmacists familiar with irAEs should be involved early, and hospitalization may be necessary in serious (≥ grade 4) or grade 3 irAEs that do not respond to therapy, or to expedite work-up and prevent complications from potentially life-threatening irAEs [28]. Patient education on the potential for irAE development is a key component of any pre-treatment discussion with patients considered suitable candidates for immunotherapy. It is also important to establish physician networks to share outcomes of successful irAE treatment strategies. Short-term adverse events due to the use of moderate to high dose corticosteroids (e.g., opportunistic infections, sleep disturbance, gastritis, and hypertension) should be anticipated. Patients receiving long-term or high dose corticosteroids are at risk of developing diabetes mellitus and osteoporosis and should receive vitamin D and calcium supplementation and, in some cases, antibiotic prophylaxis [28]. However, conflicting reports on the associated between antibiotic use and ICI efficacy pose as yet unanswered about whether routine antimicrobial prophylaxis is appropriate in patients receiving ICIs [29, 30]. For steroid-refractory cases and/or when steroid sparing is desirable, management should be coordinated with disease specialists. Other immunomodulatory agents, such as infliximab, other tumor necrosis factor inhibitors (TNFi), mycophenolate mofetil, anti-thymocyte globulin (ATG), calcineurin inhibitors, methotrexate, or intravenous immunoglobulin (IVIG) and plasmapharesis may be required. However, besides TNFi for colitis, these immunosuppressive treatments have not been evaluated in large numbers of patients. Some retrospective analyses suggest that use of corticosteroids for the management of irAEs is not associated with inferior results of therapy [31, 32] but, due to confounding, the association of irAEs with immunologic activity from immunosuppression, and with individual patient efficacy, is not clear. The effects of alternative forms of immunosuppression on the efficacy of ICIs have not yet been sufficiently studied.

As physicians, nurses and patients become aware of the value of immune-based treatments, including the synergies offered by combination immunotherapy strategies, there is a pressing need for guidance on how to recognize, report and manage irAEs that arise in the course of treatment. The Common Terminology Criteria for Adverse Events (CTCAE) [33], a descriptive lexicon of terms and adverse event severity, was developed by the National Cancer Institute (NCI) at the National Institutes of Health (NIH), with the goal of standardizing AE reporting across medical specialties. However, increasing use of immunotherapy has clarified limitations in how immune-related toxicities are addressed and classified within the current CTCAE, as well as in other databases such as the Medical Dictionary for Regulatory Activities (MedDRA). Importantly, the need for formal pathways for reporting suspected irAEs has also highlighted the tendency for CTCAE grading to under- or over-estimate true irAE incidence and/or severity [28]. In certain settings, such as with rheumatologic irAEs, CTCAE criteria are difficult to apply and do not allow accurate recording of the severity and impact of irAEs, especially as conditions may become chronic [34]. These shortcomings present an opportunity to improve and streamline irAE reporting in the next versions of CTCAE and MedDRA. Similarly, since drug labels for FDA-approved checkpoint inhibitors are based on clinical trial data for individual drugs and do not always align across therapeutic class, clinicians need multidisciplinary, broad perspective guidance on how to manage organ-specific toxicities.

To this end, the Society for Immunotherapy of Cancer (SITC) established a Toxicity Management Working Group to develop consensus recommendations on management of irAEs that develop following ICI therapy until evidence-based data are available to inform clinical decision-making. This report represents the outcome of a recent workshop to standardize toxicity management. The results represent consensus thinking by a multidisciplinary group of experts in the field but should not replace sound clinical judgment or personalized drug management, as immunotherapy patients often require highly individualized management.

## Methods

### Consensus group representation

In response to the need for a collaborative, multidisciplinary approach to the management of ICI toxicities, the SITC convened a one-day workshop on March 31st, 2017, in Washington D.C. The meeting was a multi-stakeholder effort with participation from approximately 85 experts from academia, government, industry, scientific organizations and other related entities. Representation was sought from medical oncologists, surgeons, disease subspecialists, basic scientists, pharmacists, industry clinical, regulatory and safety experts and nurses. In order to streamline recommendations across the range of organizations active in the area of cancer immunotherapy, SITC invited representatives from the American Society of Clinical Oncology (ASCO), National Comprehensive Cancer Network (NCCN), Parker Institute for Cancer Immunotherapy, Friends of Cancer Research, American Association for Cancer Research (AACR), Association of Community Cancer Centers (ACCC), NCI and the Oncology Nursing Society (ONS) to participate in the workshop. To ensure that commercial interests did not influence the outcomes of the workshop, industry representatives participated in group discussions but final approval of the workshop output, and of this manuscript, was the responsibility of the organizing committee, none of whom are employed by a pharmaceutical or biotechnology company. Representatives from the Office of Hematology and Oncology Products, Center for Drug Evaluation and Research (CDER), were invited to review and provide feedback on the final manuscript. Individuals selected as authors were workshop organizers and lead discussants for individual organ-specific toxicity breakout groups. All participants were required to disclose any potential conflicts of interest prior to participation.

### Workshop objectives and procedures

The overarching goals of the workshop were twofold: 1) to develop treatment algorithms for managing common and rare immunotherapy-related toxicities and 2) to develop standardized templates, including inclusion and exclusion criteria, for irAE management in clinical trial protocols (which will be reported separately). More broadly, participants were charged with describing the spectrum of immune-related toxicities and providing recommendations on recognizing, monitoring and managing these toxicities. To facilitate discussion among experts in different medical fields, attendees broke out into 11 subgroups (‘breakout groups’) that focused on irAEs identified by body system (dermatologic, gastrointestinal, endocrine, pulmonary, rheumatologic, cardiovascular, hematologic, renal, neurologic and ophthalmologic) as well as infusion reactions. These breakout groups were generally supplemented with disease subspecialty expertise focused on the area of interest. Each breakout group received instructions to guide their discussion, a list of recognized toxicities by system, relevant drug package inserts, several key supporting references, and a copy of CTCAE version 4.0. A working draft of the Friends of Cancer Research/Parker Institute for Cancer Immunotherapy guidelines on monitoring, management and follow-up of irAEs from anti-PD-1/PD-L1 agents was also distributed [35].

After separate breakout group discussions, one representative from each group presented their recommendations to all participants, and responded to questions and additional suggestions from the wider group. Following the meeting, recommendations made on-site were recirculated by email to participants from each breakout group to ensure all views and opinions were captured. The final recommendations on management of irAEs presented in this paper therefore represent the views of each multidisciplinary expert group. These recommendations are not intended to provide comprehensive medical guidance on the management of disorders that may arise from use of immunotherapy treatment; specialist care should be sought as necessary, and as indicated in treatment-specific guidelines.

### Strengths and limitations of the consensus recommendations

These consensus recommendations represent the views of a broad range of experts from multiple fields of expertise, and from large cancer organizations with differing areas of focus. In some cases they are driven by evidence from the published literature; in others, particularly where data are lacking, they are guided by accumulated clinical experience and practice. The participation of stakeholders from the pharmaceutical and biotechnology industries is another strength, ensuring that those involved in drug research and development are part of the discussion and that there is access to large industry-collected patient databases. However, it is important to acknowledge that evidence gaps are considerable, consensus was not reached on all issues, and many questions remain unanswered. Furthermore, not all working groups had representation from all specialist groups (oncologist, disease specialist, nurse, pharmacist). The recommendations may not take into account reimbursement restrictions that could limit access to recommended drugs for some patients. Lastly, but importantly, there was no patient representation. Finally, the recommendations addressed in this document reflect irAEs related to PD-1/PD-L1 and CTLA-4 inhibitors, and do not address toxicity that may ensue following administration of other classes of immunotherapy, including chimeric antigen receptor T cell (CAR T) therapy. It is unclear to what extent the recommendations can be generalized to immunotherapy agents other than those addressed in this manuscript, including agents in development.

## Consensus recommendations

The recommendations for managing toxicities associated with ICIs, below, represent the consensus views of participants in the 11 body system groups. Overall, irAEs are broken down into two major categories, based on the opinions of the workshop organizers regarding the frequency with which they are seen in clinical practice: frequently reported (dermatologic, gastroenterological, endocrine, respiratory, and rheumatologic/musculoskeletal) and uncommon (cardiovascular, hematologic, renal, neurologic and ophthalmologic). Infusion reactions, which are more common with mAbs based on a wildtype IgG1 backbone and less common with IgG4 antibodies, are also addressed. Within each body system, information is divided into three sections: clinical presentation and epidemiology, diagnostic evaluation, and guidance on when to refer to a disease specialist.

Management of irAEs relies heavily on corticosteroids, and other immunomodulatory agents, which should be prescribed judiciously to reduce the potential for short and long-term complications. It remains unclear whether prophylactic antibiotics should routinely be prescribed to reduce the potential for opportunistic infection in patients receiving steroids. Broadly, corticosteroid management can be approached as shown in Table 2, but treatment should be individualized depending on each patient’s medical history; co-morbidities; underlying disease status; type, number and severity of adverse events; ICI administered; and ability to tolerate corticosteroids.

**Table 2:** General guidance for corticosteroid management of immune-related adverse events

Grade of immune-related AE (CTCAE/equivalent)	Corticosteroid management	Additional notes
1	• Corticosteroids not usually indicated	• Continue immunotherapy
2	• If indicated, start oral prednisone 0.5-1 mg/kg/day if patient can take oral medication. • If IV required, start methylprednisolone 0.5-1 mg/kg/day IV • If no improvement in 2–3 days, increase corticosteroid dose to 2 mg/kg/day • Once improved to ≤grade 1 AE, start 4–6 week steroid taper	• Hold immunotherapy during corticosteroid use • Continue immunotherapy once resolved to ≤grade 1 and off corticosteroids • Start proton pump inhibitor for GI prophylaxis
3	• Start prednisone 1-2 mg/kg/day (or equivalent dose of methylprednisolone) • If no improvement in 2–3 days, add additional/alternative immune suppressant • Once improved to ≤ grade 1, start 4–6-week steroid taper • Provide supportive treatment as needed	• Hold immunotherapy; if symptoms do not improve in 4–6 weeks, discontinue immunotherapy • Consider intravenous corticosteroids • Start proton pump inhibitor for GI prophylaxis • Add PCP prophylaxis if more than 3 weeks of immunosuppression expected (>30 mg prednisone or equivalent/day)
4	• Start prednisone 1-2 mg/kg/day (or equivalent dose of methylprednisolone) • If no improvement in 2–3 days, add additional/alternative immune suppressant, e.g., infliximab • Provide supportive care as needed	• Discontinue immunotherapy • Continue intravenous corticosteroids • Start proton pump inhibitor for GI prophylaxis • Add PCP prophylaxis if more than 3 weeks of immunosuppression expected (>30 mg prednisone or equivalent/day)

Note: For steroid-refractory cases and/or when steroid sparing is desirable, management should be coordinated with disease specialists. AE, adverse event

Table 3 summarizes the recommended management of recognized irAEs across body systems.

**Table 3:** Recommended management of CTCAE-based immune-related adverse events due to immune checkpoint inhibitor (ICI) therapy

DERMATOLOGIC			Specialist referral?
Maculopapular rash/dermatitis			
Grade	Description	Management	
1	Macules/papules covering <10% BSA with or without symptoms (e.g., pruritus, burning, tightness)	• Continue ICI • Oral antihistamines ○ Cetirizine/loratidine 10 mg daily (non-sedating); hydroxyzine 10-25 mg QID, or at bedtime • Topical corticosteroids ○ Class I topical corticosteroid (clobetasol propionate, halobetasol propionate, betamethasone dipropionate cream or ointment) for body; Class V/VI corticosteroid (aclometasone, desonide, hydrocortisone 2.5% cream) for face	
2	Macules/papules covering 10–30% BSA with or without symptoms (e.g., pruritus, burning, tightness); limiting instrumental ADL	• Continue ICI • Non-urgent dermatology referral • Oral antihistamines ○ Cetirizine/loratidine 10 mg daily (non-sedating); hydroxyzine 10-25 mg QID, or at bedtime • Topical corticosteroids (see grade 1) ○ As above ○ Cetirizine/loratidine 10 mg daily (non-sedating); hydroxyzine 10-25 mg QID, or at bedtime	**✓**
3	Macules/papules covering >30% BSA with or without associated symptoms; limiting self-care ADL	• Hold ICI • Same day dermatology consult • Rule out systemic hypersensitivity: CBC with differential, CMP • Oral antihistamines ○ Cetirizine/loratidine 10 mg daily (non-sedating); hydroxyzine 10-25 mg QID, or at bedtime • Systemic corticosteroids • Prednisone 0.5 – 1 mg/kg/day (or equivalent dose of methylprednisolone) until rash resolves to ≤ grade 1	**✓**
Pruritus*			
Grade	Description	Management	
1	Mild or localized; topical intervention indicated	• Emollients with cream or ointment based, fragrance-free products ○ Class I topical corticosteroid (clobetasol propionate, halobetasol propionate, betamethasone dipropionate) for body; Class V/VI corticosteroid (aclometasone, desonide, hydrocortisone 2.5%) for face, AND oral antihistamines (e.g., cetirizine/loratidine 10 mg daily, hydroxyzine 10-25 mg QID, or at bedtime	
2	Intense or widespread; intermittent; skin changes from scratching (e.g., edema, papulation, excoriation, lichenification, oozing/crusts); oral intervention indicated; limiting instrumental ADL	• Dermatology referral • Class I topical steroid (clobetasol propionate, halobetasol propionate, betamethasone dipropionate) for body; class V/VI steroid (aclometasone, desonide, hydrocortisone 2.5%) for face, AND oral antihistamines (e.g., cetirizine/loratidine 10 mg daily, hydroxyzine 10-25 mg QID, or at bedtime • Oral corticosteroids ○ Prednisone 0.5 – 1 mg/kg/day (or equivalent of methylprednisolone) tapered over 2 weeks	**✓**
3	Intense or widespread; constant; limiting self-care ADL or sleep; oral corticosteroid or immunosuppressive therapy indicated	• Dermatology referral • GABA agonist (pregabalin, gabapentin 100-300 mg TID) • Oral corticosteroid ○ Prednisone 0.5 – 1 mg/kg/day (or equivalent of methylprednisolone) tapered over 2 weeks	**✓**
Notes: 1. Grade 4 maculopapular rash/dermatitis is not included in CTCAE *Recommendations provided are based on case reports, series and expert consensus. Use of suggested therapies must be discussed with medical oncology based on individual patient considerations. The impact of these therapies on the anti-tumor immune response and efficacy of cancer treatment is unknown and requires further research.			
GASTROENTEROLOGICAL			Specialist referral?
Colitis			
Grade	CTCAE description	Management	
1	Asymptomatic; clinical or diagnostic observations only; intervention not indicated [Grade 1 diarrhea frequency ≤ 4/day]	• Close follow up within 24–48 h for changes or progression • Continue ICI • If symptoms persist, start routine stool and blood tests • Bland diet advisable during period of acute diarrhea • Anti-diarrheal medication is optional but not highly recommended when infectious work-up is negative.	
2	Abdominal pain; mucus or blood in stool [Grade 2 diarrhea frequency 4–6/day]	• Hold ICI • Outpatient stool and blood work; CRP, ESR, fecal calprotectin, lactoferrin, imaging and endoscopy are optional • If diarrhea only, observe for 2–3 days. If no improvement start prednisone 1 mg/kg/day (or equivalent dose of methylprednisolone); anti-diarrheal medication is not recommended • If diarrhea and colitis symptoms (abdominal pain +/− blood in BM), start prednisone 1 mg/kg/day (or equivalent dose of methylprednisolone)immediately ○ If no improvement in 48 h, increase corticosteroid dose to prednisone 2 mg/kg/day (or equivalent dose of methylprednisolone) ○ If patient improves ▪ Taper corticosteroid over 4–6 weeks may be needed ▪ Resume ICI when corticosteroid is tapered to ≤10 mg/day and patient remains symptom-free (grade ≤ 1)* ▪ Continue anti-PD-1 or anti-PD-L1 monotherapy ▪ If using combination anti-CTLA-4/anti-PD-1 immunotherapy, continue anti-PD-1 agent only ▪ ICI dose reduction is not recommended • If colitis returns on resuming ICI: ○ Grade ≤ 2: temporarily hold ICI ○ Grade ≥ 3: permanently discontinue ICI	**✓** See note 5
3 and 4	Grade 3: Severe abdominal pain; change in bowel habits; medical intervention indicated; peritoneal signs [Grade 3 diarrhea frequency ≥ 7×/day] Grade 4: Life-threatening consequences; urgent intervention indicated	• Grade 3: withhold ICI; consider resuming ICI when corticosteroid is tapered to ≤10 mg/day and patient remains symptom-free (grade ≤ 1). Consider hospitalization • Grade 4: permanently discontinue ICI and hospitalize • Blood and stool infection work-up, inflammatory markers, imaging, endoscopy and GI consult • Start intravenous prednisone 1-2 mg/kg/day (or equivalent dose of methylprednisolone) immediately ○ If patient improves, follow instructions for ‘If patient improves’ for grade 2 • If refractory or no improvement on IV corticosteroid, start prednisone 2 mg/kg/day (or equivalent dose of methylprednisolone) for 3 days • Consider other anti-inflammatory agents e.g. infliximab 5 mg/kg, which can be given again after two weeks if a second dose is needed. Vedolizumab may also be used (see Note 4 below).	
Notes: 1. CBC with differential, CMP, ESR and CRP are recommended before starting immunotherapy, to provide baseline values for comparison over time. Despite the association between elevated ESR and CRP and colitis, some insurance companies may not cover these tests. 2. There is no proven role for prophylactic corticosteroids (budesonide) to prevent GI irAEs [45, 47]. 3. Response to infliximab generally occurs within 1–3 days although some patients benefit from a second dose after 2 weeks. Prolonged oral prednisone taper may be required after infliximab administration. Whether infliximab reduces the antitumor efficacy of ipilimumab remains unknown [103]. 4. Case reports of successful treatment of steroid-dependent immune-related colitis using vedolizumab indicate this may benefit certain patients. 5. A GI consult is warranted in any patient who meets criteria for grade 2 diarrhea/colitis with negative infectious stool work up.			
Hepatitis			
Grade	CTCAE Description (Note 1)	Management	
1	AST, ALT > ULN -3xULN; total bilirubin > ULN-1.5xULN	• Continue ICI • CMP or hepatic function panel once weekly • If liver enzyme and function tests are stable, reduce frequency of blood tests	
2	AST, ALT >3- ≤ 5xULN; total bilirubin >1.5 - ≤ 3xULN	• Hold ICI • Rule out viral hepatitis, autoimmune disease, biliary obstruction, new metastasis or thrombosis • Start prednisone 0.5-1 mg/kg/day (or equivalent dose of methylprednisolone) with 4 week taper • Monitor CMP twice a week • Liver biopsy is optional • Resume ICI when corticosteroid taper to 10 mg/day (toxicity grade ≤ 1)	
3 and 4	AST, ALT >5xULN; total bilirubin >3xULN	• Permanently discontinue ICI • Monitor CMP every 1–2 days • Start prednisone 1–2 mg/kg/day ○ If refractory after 3 days, consider mycophenolate • If liver enzymes improve, taper corticosteroid over 4 weeks • Consider liver biopsy	
Notes: 1. Liver enzyme levels stated here are not defined in CTCAE and are instead drawn from reference [104] 2. In patients with liver metastasis, ICI can be used at baseline liver profile equivalent to grade 2. If ≥50% elevation in AST/ALT lasting for ≥1 week, permanently stop ICI.			
ENDOCRINE			Specialist referral?
Hypophysitis			
Grade	CTCAE Description*	Management	
1	Asymptomatic or mild symptoms; clinical or diagnostic observations only; intervention not indicated	• Hold ICI if ≥ grade 2 irAE until work up is completed and appropriate hormone replacement is started • If central adrenal insufficiency: start physiologic steroid replacement: Hydrocortisone ~10 mg/m^2^ (HC 15 mg am, 5 mg at 3 pm) ○ Periodic assessment (e.g., every 3 months in the first year, every 6 months thereafter): clinical monitoring and repeat hormone levels (am cortisol and ACTH and/or low dose cosyntropin stimulation test) to assess recovery • If central hypothyroidism: start thyroid hormone (levothyroxine 1mcg/kg) ○ Repeat thyroid function testing 6–8 weeks after initiation of thyroid hormone and then periodically (e.g., every 3 months in the first year and every 6 months thereafter) to assess recovery • If central hypogonadism, repeat hormone levels in 2–3 months and consider testosterone in men or HRT in women if appropriate for cancer type *For severe/life-threatening symptoms such as adrenal crisis, severe headache, visual field deficiency:* • Hospitalize as appropriate. • High dose corticosteroid (prednisone 1 mg/kg/day) (or equivalent dose of methylprednisolone) in the acute phase, followed by taper over 1 month. • Adrenal crisis should be managed per standard guidelines. • If central hypothyroidism, replace thyroid hormone (see above) after corticosteroids have been initiated	**✓**
2	Moderate; minimal, local or noninvasive intervention indicated; limiting age- appropriate instrumental ADL		
3	Severe or medically significant but not immediately life-threatening; hospitalization or prolongation of existing hospitalization indicated; disabling; limiting self-care ADL		
4	Life-threatening consequences; urgent intervention indicated		
Note: In the uncommon scenario of MRI findings without pituitary deficiency, consider high dose corticosteroids for prevention of hormonal dysfunction. * Hypophysitis is not defined in CTCAE Version 4.0. This classification is drawn from the CTCAE category ‘Endocrine disorders – Other’.			
Hypothyroidism			
Grade	CTCAE Description	Management	
1	Asymptomatic; clinical or diagnostic observations only; intervention not indicated	• Hold ICI for ≥grade 3 irAEs • ICI can be continued after resolution of symptoms to grade 2 or better. • Start standard thyroid replacement therapy: initial dose can be the full dose (1.6 mcg/kg) in young, healthy patients, but a reduced dose of 25 -50mcg should be initiated in elderly patients with known cardiovascular disease. • Repeat TSH and free T4 testing after 6–8 weeks and adjust thyroid hormone dose accordingly. If TSH is above reference range, increase thyroid hormone dose by 12.5 mcg to 25 mcg • After identification of the appropriate maintenance dose, further evaluation is required every year, or sooner if patient’s status changes • After identification of the appropriate maintenance dose, further evaluation is required every year, or sooner if patient’s status changes	**✓**
2	Symptomatic; thyroid replacement indicated; limiting instrumental ADL		
3	Severe symptoms; limiting self-care ADL; hospitalization indicated		
4	Life-threatening consequences; urgent intervention indicated		
Hyperthyroidism			
Grade	CTCAE Description	Management	
1	Asymptomatic; clinical or diagnostic observations only; intervention not indicated	• Hold ICI for ≥ grade 3 irAEs • Standard therapy for hyperthyroidism should be followed • *Thyroiditis* is self-limiting and has 2 phases: ○ In the hyperthyroid phase*,* patients may benefit from beta blockers if symptomatic (e.g., atenolol 25–50 mg daily, titrate for HR < 90 if BP allows). Monitor closely with regular symptom evaluation and free T4 testing every 2 weeks. ○ Introduce thyroid hormones (see hypothyroidism management) if the patient becomes hypothyroid (low free T4/T3, even if TSH is not elevated). • *Graves’ disease* should be treated per standard guidelines.	**✓**
2	Symptomatic; thyroid suppression therapy indicated; limiting instrumental ADL		
3	Severe symptoms; limiting self-care ADL; hospitalization indicated		
4	Life-threatening consequences; urgent intervention indicated		
Note: High dose corticosteroids (1 mg/kg/day) are not routinely required.			
Type 1 diabetes (CTCAE defines hyperglycemia not diabetes)			
Grade	CTCAE Description	Management	
1	Fasting glucose > ULN - 160 mg/dL (>ULN - 8.9 mmol/L)	• *Type 1 DM with diabetic ketoacidosis*: Hold ICI; hospitalize and initiate treatment per standard guidelines. • *Type 1 DM without diabetic ketoacidosis*: Hold ICI for hyperglycemia ≥ grade 3. Treat with insulin and continue ICI when patient recovers to grade 1. • Treat with insulin per standard guidelines and restart ICI when patient recovers to grade 1. • Provide patient education on diet and lifestyle modification, and blood glucose testing	**✓**
2	Fasting glucose >160–250 mg/dL (>8.9–13.9 mmol/L)		
3	Fasting glucose >250–500 mg/dL (>13.9–27.8 mmol/L); hospitalization indicated		
4	Fasting glucose >500 mg/dL (>27.8 mmol/L); life-threatening consequences		
PULMONARY			Specialist referral?
Pneumonitis			
Grade	CTCAE Description	Management	
1	Asymptomatic; clinical or diagnostic observations only	• Consider holding ICI • Consider pulmonary and infectious disease consultations • Reimage at least prior to every cycle of ICI treatment (at least every 3 weeks) ○ If repeat imaging shows resolution of radiographic findings, no further CT imaging is necessary; resume therapy with close follow-up ○ If evidence of progression, treat at higher grade ○ If no change, consider continued therapy with close follow-up for new symptoms • If symptoms develop, treat at higher grade • Self-monitor symptoms and oxygen saturation (using personal pulse oximeter) every 2–3 days; weekly clinic visits • If chest imaging abnormalities resolve, consider resuming treatment with close follow-up	**✓**
2	Symptomatic; limiting instrumental ADL; medical intervention indicated	• Hold ICI • Consider hospitalization • Pulmonary consultation for bronchoscopy with bronchoalveolar lavage. Consider biopsies for atypical lesions • Initiate methylprednisolone 1 mg/kg/day (IV or oral equivalent) ○ Day 2–3 of corticosteroids/supportive care: If symptoms improve to ≤ grade 2, start slow steroid taper over >1 month. If symptoms do not improve, or worsen, treat as grade 3–4 • Consider drug re-challenge if symptoms and imaging abnormalities resolve	**✓** (pulmonary and infectious disease)
3	Severe symptoms; limiting self-care ADL; oxygen indicated	• Permanently discontinue ICI • Hospitalize; consider ICU care • Pulmonary consultation for bronchoscopy with bronchoalveolar lavage. Consider biopsies for atypical lesions • Initiate methylprednisolone IV, 2 mg/kg/day • Day 2–3 of corticosteroids/supportive care: ○ If no clinical improvement, add infliximab or cyclophosphamide, mycophenolate mofetil or IVIG ○ If clinical improvement: reduce corticosteroids to 1 mg/kg/day and slowly taper over >2 months. • Drug re-challenge: ○ Grade 3: Consider drug re-challenge on a case-by-case basis after discussions weighing risk/benefit with the patient and only if symptoms and imaging abnormalities resolve ○ Grade 4: Permanent y discontinue ICI	✓ (pulmonary and infectious disease)
4	Life-threatening respiratory compromise; urgent intervention indicated (e.g., intubation)		
Notes: 1. Consider prophylactic antibiotics for pneumocystis pneumonia (PCP) for patients receiving at least 20 mg methylprednisolone or equivalent for ≥4 weeks 2. Consider calcium and vitamin D supplementation with prolonged steroid use 3. All patients with grade 2–4 pneumonitis receiving steroids should also be on proton pump inhibitor therapy for GI prophylaxis 4. T-spot testing should be undertaken to exclude tuberculosis in any patient being considered for anti-TNF therapy, prior to starting anti-TNF treatment.			
Sarcoidosis			
Grade	CTCAE Description	Management	
1	Not defined in CTCAE	• Consider holding ICI • Close follow-up • Consider corticosteroids • Hold ICI • Consider corticosteroid therapy for patients with sarcoidosis grade 2 or higher and any of the following: ○ progressive radiographic change ○ persistent and/or troublesome pulmonary symptoms ○ lung function deterioration: TLC decline of ≥10%, FVC decline of ≥15%; DLCO decline of ≥20% ○ concomitant involvement of critical extrapulmonary organ systems ○ sarcoid-related hypercalcemia • Corticosteroid dose: prednisone 1 mg/kg (or IV equivalent of methylprednisolone) for grade 2 sarcoidosis or severe cases requiring hospitalization. Taper steroids over 2–4 months, depending on response	**✓** **✓**
≥ 2			
Notes: To date, there are no studies focusing on management of sarcoidosis as a side effect of checkpoint inhibitor therapy. Current recommendations are based on clinical experience and case report publications.			
RHEUMATOLOGIC/MUSCULOSKELETAL [78]			Specialist referral?
Inflammatory arthritis			
Grade	CTCAE Description (Note 1)	Management	
1	Mild pain with inflammatory symptoms (Note 2), erythema, or joint swelling (Note 3)	• Continue ICI • Analgesics: NSAIDs: naproxen 500 mg BID or meloxicam 7.5–15 mg daily orally for 4–6 weeks • If NSAIDs ineffective, consider prednisone 10–20 mg daily for 2–4 weeks • Consider intra-articular corticosteroid injection only if ≤2 joints affected and low dose prednisone (10 mg/day) and NSAIDs not effective • If no improvement in 2–4 weeks, escalate to grade 2 management • Conduct serial rheumatologic examinations (2 weeks, 4 weeks, then 4–6 weekly) and functional assessment at follow-up	
2	Moderate pain associated with signs of inflammation, erythema, or joint swelling; limiting instrumental ADL	• Consider holding ICI • Rheumatology referral to confirm inflammatory arthritis, assess need for intra-articular injection and examine for signs of early bone damage • Prednisone 20 mg daily for 2–4 weeks, increase to 1 mg/kg/day, or equivalent. If no response in 2–4 weeks. Escalate to grade 3 management • If symptoms improve, taper corticosteroid over 4–8 weeks or until grade 1	**✓**
3	Severe pain associated with signs of inflammation, erythema, or joint swelling; irreversible joint damage (e.g., erosion); disabling; limiting self-care ADL	• Hold ICI • Rheumatology referral • Prednisone 1 mg/kg/day or equivalent for 2–4 weeks, or until symptoms improve to grade 1 • Consider additional immunosuppression (Note 4) (e.g. methotrexate [Note 5], sulfasalazine, leflunomide). Consider anti-cytokine therapy (e.g. TNF-inhibition) [Note 6] • If symptoms improve, taper corticosteroid over 4–8 weeks/until grade 1; if symptoms do not improve in 4–6 weeks: permanently discontinue ICI	**✓**
Notes: 1. CTCAE includes separate listings for arthritis, joint effusion and arthralgia although there is overlap in presenting symptoms such as pain and effects on ADL 2. Joint stiffness after sleep or inactivity, improvement of symptoms with movement or heat. 3. Joint swelling refers to the clinical finding on examination, and may encompass soft tissue swelling, joint effusion or synovitis. 4. Before initiation of these drugs, screening for hepatitis B and C should be performed 5. Methotrexate should be administered at a starting dose of 15 mg weekly, with daily folic acid supplementation. Titrate up to a maximum of 25 mg weekly, or switch to injectable methotrexate if patient cannot tolerate orally 6. Before anti-cytokine therapy, evaluation for latent/active TB should be performed			
INFUSION REACTIONS			Specialist referral?
Grade	CTCAE Description	Management	
1	Mild transient reaction; infusion interruption not indicated; intervention not indicated	• Drug infusion rate may be decreased, or infusion temporarily interrupted, until resolution of the event • Consider reducing the rate of infusion upon re-initiation or subsequent infusions • Non-steroidal anti-inflammatory drugs (NSAIDs, e.g. acetaminophen), antihistamines, opioids, and corticosteroids may be used per investigator/ institutional guidelines • Consider premedication for subsequent infusions per investigator/ institutional guidelines	
2	Therapy or infusion interruption indicated but responds promptly to symptomatic treatment (e.g., antihistamines, NSAIDS, narcotics, IV fluids); prophylactic medications indicated for ≤24 h.		
3	Prolonged (e.g., not rapidly responsive to symptomatic medication and/or brief interruption of infusion); recurrence of symptoms following initial improvement; hospitalization indicated for clinical sequelae	• Permanently discontinue ICI • For severe/life-threatening reactions, manage the patient as clinically appropriate (e.g. antihistamines, oxygen, fluids, opioids, corticosteroids, bronchodilators, etc.) per investigator/ institutional guidelines	**✓**Refer to allergist to prevent potential future reactions
4	Life-threatening consequences; urgent intervention indicated		
CARDIOVASCULAR			Specialist referral?
Grade	CTCAE Description	Management	
1	Abnormal cardiac biomarker testing, including abnormal ECG	• Recommend baseline ECG and cardiac biomarker assessment (BNP, troponin) to establish if there is a notable change during therapy • Mild abnormalities should be observed closely during therapy	**✓** if abnormal
2	Abnormal screening tests with mild symptoms	• Control cardiac diseases (e.g. heart failure, atrial fibrillation) optimally • Control cardiac disease risk factors proactively (including hypertension, hyperlipidemia, discontinue smoking, and monitor diabetes)	**✓**
3	Moderately abnormal testing or symptoms with mild activity	• BNP > 500 pg/ml, troponin >99% institutional normal, new ECG findings (QTc prolongation, new conduction disease, or ST-T wave changes) • Consider withholding ICI ○ If a period of stabilization is achieved and definite cardiac toxicity was not identified, it may be reasonable to consider re-challenging the patient with ICI, with heightened monitoring. • If confirmed cardiac injury or decompensation, hold ICI therapy until stabilized. • Optimally treat identified cardiac conditions • Consider corticosteroids if myocarditis suspected (Note 2)	**✓**
4	Moderate to severe decompensation, intravenous medication or intervention required, life threatening conditions	• Permanently discontinue ICI • If myocarditis is identified, consider high-dose corticosteroids (1 mg/kg methylprednisolone (IV) for at least several days) until improved to grade ≤ 1, after that consider at least 4–5 weeks of tapering doses (Note 2). • Add additional immunosuppressive agents in severe refractory cases. • Give additional supportive treatments, including appropriate treatment of heart failure. Additional treatment of detected cardiac conditions should be provided.*	**✓**
Notes: 1. Grades outlined here are not drawn from CTCAE. 2. Patients with confirmed myocarditis (or in cases of reasonable suspicion) should receive emergent high-dose corticosteroids. Until data are available (e.g., cut-off levels of troponin) to determine when to start corticosteroids in patients with possible (as opposed to confirmed) myocarditis, this decision should be made on a case by case basis. The importance of active, ongoing consultation with a cardiologist to discuss the risk/benefit of continuing ICI therapy, starting corticosteroids, or instituting other cardiac treatments, cannot be overstated. * Other therapies for management of myocarditis or pericarditis (viral based therapy, immunoglobulins, or plasmapheresis) are speculative at this point in time.			
HEMATOLOGIC			Specialist referral?
Anemia			
Grade	CTCAE Description	Management	
1	Hgb < LLN - 10.0 g/dL; <LLN - 6.2 mmol/L; <LLN - 100 g/L	• Monitor closely while continuing ICI	
2	Hgb <10.0–8.0 g/dL; <6.2–4.9 mmol/L; <100 - 80 g/L	• Monitor closely while continuing ICI • Evaluate for possible causes and refer to hematology if no obvious cause if identified	**✓** if no cause identified
3	Hgb <8.0 g/dL; <4.9 mmol/L; <80 g/L; transfusion indicated	• Hold ICI • Consider Coombs testing and evaluation for hemolytic anemia • Consider re-treating with ICI if hemolytic anemia responds promptly (within a few days) to corticosteroids	**✓**
4	Life-threatening consequences; urgent intervention indicated	• Permanently discontinue ICI	**✓**
Notes: 1. No firm recommendations for corticosteroid management are provided here as treatment should be individualized. 2. If unexplained anemia does not respond to steroids, consider bone marrow biopsy.			
Thrombocytopenia (CTCAE defines decreased platelet count not thrombocytopenia)			
Grade	CTCAE Description	Management	
1	<LLN - 75,000/mm3; <LLN-75.0 x 10e9 /L	• *Progressive or grade 3 unexplained thrombocytopenia*: consider work up for autoimmune disease and rule out DIC or other cause of thrombocytopenia that may be related to underlying disease • *Precipitous development of thrombocytopenia*: consider steroid intervention pending clinical condition (brain metastases, colitis, etc.) and evaluate for immune-mediated thrombocytopenia • Permanently discontinue ICI for clinically significant, steroid-refractory ICI-associated thrombocytopenia	
2	<75,000–50,000/mm3; <75.0–50.0 x 10e9 /L		**✓** if no cause identified
3	<50,000–25,000/mm3; <50.0–25.0 x 10e9 /L		**✓**
4	<25,000/mm3; <25.0 x 10e9 /L		**✓**
Note: No firm recommendations for corticosteroid management are provided here as treatment should be individualized.			
RENAL			Specialist referral?
Nephritis			
Grade	CTCAE Description	Management	
1	Creatinine level increase of >0.3 mg/dL; creatinine 1.5–2.0× above baseline	• Continue ICI but initiate work-up to evaluate possible causes and monitor closely	
2	Creatinine 2 - 3× above baseline	• Hold ICI ○ Resume when creatinine decreased to ≤grade 1 (Note 2) • Consider timing of event and response to treatment when making a decision • Start corticosteroids (Note 3) • Discontinue ICI for persistent or recurrent elevation	
3	Creatinine >3 x baseline or >4.0 mg/dL; hospitalization indicated	• Hold ICI • Consider resuming treatment if grade 3 resolves (Note 2) and cause of event is confirmed. Timing of event and response to treatment should be considered in making a decision • Start corticosteroids (Note 3) • Discontinue ICI for persistent or recurrent elevation	
4	Life-threatening consequences; dialysis indicated	• Permanently discontinue ICI • Start corticosteroids (Note 3)	**✓**
Notes: 1. Grades are those listed under ‘acute kidney injury’ in CTCAE [33]. 2. Consider using increase from baseline rather than absolute value for creatinine monitoring, especially in patients with primary renal carcinoma or other baseline renal conditions. 3. For persistent creatinine elevation ≥ grade 2 with no other identifiable cause, start corticosteroids. Dose and schedule should be individualized and based on grade. Taper corticosteroids when creatinine improves to grade 1.			
NEUROLOGIC			Specialist referral?
Encephalopathy/Leukoencephalopathy/Reversible posterior leukoencephalopathy syndrome (PRES)			
Grade	CTCAE Description	Management	
1	Mild symptoms	• Hold ICI and initiate diagnostic work-up • Consider permanent discontinuation of ICI if AE worsens or does not improve	
2	Moderate symptoms; limiting instrumental ADL	• Hold ICI • Start 0.5–1.0 mg/kg/day methylprednisolone equivalents PO or IV once infection has been excluded • Consider permanent discontinuation of ICI if AE worsens or does not improve.	**✓**
3	Severe symptoms; limiting self-care ADL	• Permanently discontinue ICI • Start 1–2 mg/kg/day methylprednisolone equivalents IV and prophylactic antibiotics • Consider plasmapheresis if no improvement or symptoms worsen after 3 days	**✓**
4	Life-threatening consequences; urgent intervention indicated	• Permanently discontinue ICI • Start 1–2 mg/kg/day methylprednisolone equivalents IV and prophylactic antibiotics • Consider plasmapheresis if no improvement or symptoms worsen after 3 days • Contact intensive care unit	**✓** and contact intensive care unit
Notes: CTCAE provides grading criteria for encephalopathy, leukoencephalopathy, and reversible posterior leukoencephalopathy syndrome (PRES). For all these irAEs, ICI therapy may be continued for grade 1 irAEs. However, ≥ grade 2 events require an ICI hold, and referral to neurology. For events of ≥ grade 3 severity, ICI should be permanently discontinued, IV corticosteroids administered, and plasmapheresis considered if there is no improvement, or symptoms worsen, after 3 days.			
Peripheral motor and sensory neuropathy			
Grade	CTCAE Description	Management	
1	See CTCAE for grade definitions for each disorder	• Continue ICI • Consider permanent discontinuation of ICI if AE worsens or does not improve	
2		• Hold ICI • Refer to neurology • Consider permanent discontinuation of ICI if AE worsens or does not improve	**✓**
3		• Permanently discontinue ICI • Start 1–2 mg/kg/day methylprednisolone equivalents IV, and prophylactic antibiotics	**✓**
4			
Notes: CTCAE provides grading criteria for peripheral motor neuropathy and sensory motor neuropathy. For all these irAEs, ICI therapy may be continued for grade 1 irAEs. However, ≥ grade 2 events require an ICI hold and referral to neurology. For events of ≥ grade 3 severity, ICI therapy should be permanently discontinued and IV corticosteroids administered.			
OPHTHALMOLOGIC			
Uveitis			
Grade	CTCAE Description	Management	
1	Asymptomatic; clinical or diagnostic observations only	• Continue ICI • Ophthalmology referral within 1 week • Start lubrication drops (artificial tears)	**✓**
2	Anterior uveitis; medical intervention indicated	• Hold ICI • Ophthalmology referral within 2 days, prior to initiating uveitis treatment • Coordinate treatment with ophthalmologist (topical corticosteroids, cycloplegic agents, systemic corticosteroids)	**✓**
3	Posterior or pan-uveitis (Note 1)	• Permanently discontinue ICI • In carefully selected cases it may be appropriate to restart treatment, cautiously, depending on severity, systemic response to immunotherapy and ocular response to topical, local or systemic prednisone (prescribed in coordination with ophthalmologist) • URGENT ophthalmology referral (preferably uveitis specialist) prior to initiating treatment. Co-ordinate treatment with specialists • Consider systemic corticosteroids in addition to intravitreal/periocular corticosteroids/topical corticosteroid treatment as recommended by ophthalmologist	**✓** URGENT
4	Blindness (20/200 or worse) in the affected eye	• Permanently discontinue ICI • URGENT ophthalmology referral (preferably uveitis specialist) prior to initiating any treatment. Co-ordinate treatment with specialists • Consider systemic corticosteroids in addition to intravitreal /periocular corticosteroids/topical corticosteroid treatment as recommended by ophthalmologist	**✓** URGENT
Note: Unlike anterior uveitis, posterior uveitis can be asymptomatic but nonetheless proceed to visual loss.			
Episcleritis			
Grade	CTCAE Description	Management	
1	Asymptomatic; clinical or diagnostic observations only	• Continue ICI • Ophthalmology referral within 1 week • Start lubrication drops (artificial tears)	**✓**
2	Symptomatic, limiting instrumental ADL; moderate decrease in visual acuity (20/40 or better)	• Hold ICI • Ophthalmology referral within 2 days, prior to initiating uveitis treatment • Coordinate treatment with ophthalmologist (topical steroids, cycloplegic agents, systemic steroids) (See Note)	**✓**
3	Symptomatic, limiting self- care ADL; marked decrease in visual acuity (worse than 20/40)	• Permanently discontinue ICI • In carefully selected cases it may be appropriate to restart treatment, cautiously, depending on severity, systemic response to immunotherapy and ocular response to topical, local or systemic prednisone (prescribed in coordination with ophthalmologist) • URGENT ophthalmology referral (preferably uveitis specialist) prior to initiating treatment (See Note). Co-ordinate treatment with specialists. • Consider systemic steroids in addition to intravitreal /periocular steroids /topical steroid treatment as recommended by ophthalmologist	**✓** URGENT
4	Blindness (20/200 or worse) in the affected eye	• Permanently discontinue ICI • URGENT ophthalmology referral (preferably uveitis specialist) prior to initiating any treatment (See Note). Co-ordinate treatment with specialists. • Consider systemic steroids in addition to intravitreal /periocular steroids /topical steroid treatment as recommended by ophthalmologist	**✓** URGENT
Notes: IMPORTANT: Starting treatment with steroids prior to conducting an eye exam may worsen ocular conditions that are due to infection (e.g., herpetic keratitis/uveitis) or may mask accurate diagnosis and severity grading when the patient is examined by an ophthalmologist.			
Blepharitis			
Grade	CTCAE Diagnosis	Management	
Not defined in CTCAE		• Puffy eyelids may indicate early preseptal cellulitis, which requires systemic antibiotic treatment. Warning signs (eyelid swelling with pain and erythema, proptosis, pain with eye movements, movement restriction/diplopia, vision changes) should prompt urgent ophthalmology referral • In the absence of warning signs, start warm compresses and lubrication drops and refer to ophthalmology, especially if symptoms do not improve	**✓** URGENT if warning signs

[Note: Recommended management of uncommon dermatologic immune-related adverse events is presented in Additional file 1: Table S1]

### Frequently reported immune-related adverse events

#### Dermatologic adverse events

##### Clinical presentation and epidemiology

Maculopapular rash and pruritus are common reactions to ICIs but lichenoid, eczematous, and bullous dermatitis, and psoriasis have also been reported, albeit less frequently. Vitiligo is frequently seen in the melanoma patient population. Dermatologic toxicity (all grades) is reported in 30–40% of patients taking PD-1/PD-L1 inhibitors [13, 15], and approximately 50% of patients treated with ipilimumab [13]. A systematic review of the literature reported that 13–20% of patients taking pembrolizumab or nivolumab developed rash or pruritus (all-grade) and approximately 8% (all with melanoma) developed vitiligo [36], which is associated with tumor response [20]. More recently, several cases of hair re-pigmentation have also been described in patients treated with anti-PD1 or anti-PD-L1 therapy [37]. Onset of skin irAEs typically occurs within days or weeks of treatment [38] although onset may be delayed, appearing after several months of treatment [39]. Most dermatologic irAEs are low-grade and manageable, [13, 36] although rare, potentially life-threatening exfoliative dermatological conditions such as Stevens-Johnson Syndrome/toxic epidermal necrolysis (SJS/TEN), and drug rash with eosinophilia and systemic symptoms (DRESS) have been reported [28]. Severe irAEs tend to occur more commonly with combination ICI therapy [40]. Any clinical suspicion of such reactions should prompt immediate specialist referral. Permanent discontinuation of immunotherapy is mandatory for grade 4 dermatologic irAEs, SJS/TEN, or DRESS syndrome.

##### Diagnostic evaluation:

Given the frequency and persistence of skin toxicities with ICIs, dermatologic assessments are warranted in patients with a known history of immune-related skin disorders such as psoriasis, bullous pemphigoid or lupus. Non-specific maculopapular eruptions are commonly reported, which may, in part, reflect the limitations of CTCAE in the classification of specific subsets of skin disorders. Whenever possible, the irAE should be categorized since management algorithms reflect the approach to idiopathic skin disorders, beyond systemic immune suppression with steroids. Patients should undergo full skin and mucosal exam, taking note of the extent and type of lesions present.

##### When to refer

In cases of non-urgent or emergent referral, photographic documentation is recommended when a new dermatologic manifestation appears, prior to implementing treatment. This facilitates later classification of the AE when necessary. A same-day dermatology consult is warranted in any patient with blisters covering ≥1% body surface area (BSA), a rash with mucosal involvement, any rash covering ≥30% BSA, and rash with skin pain with or without blisters (excluding dermatomal varicella zoster). For these latter cases, skin biopsy is recommended to help classify the event. Non-acute dermatology referral is recommended for rashes where diagnosis is unclear, grade 2 rash that is worsening, erythema multiforme, blistering disorders of any BSA or for a rash consistent with psoriasis or lichenoid dermatitis that has not responded to topical intervention. Any grade 3 dermatologic toxicity warrants a same-day dermatology consult. Patients with suspected SJS/TEN, severe mucocutaneous reactions characterized by epidermal necrosis and detachment, should be hospitalized immediately and a dermatologist consulted for administration of systemic immunosuppression.

The recommended management of common dermatologic irAEs is presented in Table 3; recommendations for managing uncommon dermatologic irAEs is presented in Additional file 1:Table S1.

## Gastrointestinal adverse events

### Clinical presentation and epidemiology

#### Colitis

Diarrhea is one of the most frequently reported irAEs in patients taking ICIs. Mild, transient, self-limited diarrhea that occurs on initiation of an immune response should be distinguished from other presentations. Onset occurs after an average of three infusions [11], although it may occcur as soon as following the first infusion. Incidence is higher among patients taking combination anti-CTLA-4/anti-PD-1 therapy (44%) than those receiving anti-CTLA-4 (23–33%) or anti-PD-1 (≤19%) monotherapy. The combinatorial approach is also associated with increased risk of grade 3/4 symptoms compared with monotherapy, and the proportion of patients experiencing high-grade symptoms is greater with ipilimumab than anti-PD-1 or anti-PD-L1 agents [15, 40, 41]. The presence of diarrhea in conjunction with abdominal pain, rectal bleeding, mucus in the stool, and fever should alert the clinician to the possibility of colitis, a potentially serious or even life-threatening gastrointestinal (GI) complication of ICI therapy. Reports differ on the primary location of ICI colitis, with some finding a uniform distribution [42], and others observing that inflammation preferentially affects the descending colon [43, 44], although this may be due to less frequent examination of the proximal colon [44, 45]. Diarrhea and/or colitis may recur months after discontinuation of immunotherapy and can mimic chronic inflammatory bowel disease (IBD) [42, 46].

#### Hepatitis

Less frequently observed, but nonetheless well-recognized in patients treated with ICIs, is a typically asymptomatic immune-related hepatitis characterized by elevated alanine aminotransferase (ALT) or aspartate aminotransferase (AST), with or without raised bilirubin. Median onset of transaminase elevation is approximately 6–14 weeks after starting ICI treatment [28]. A minority of patients present with fever. The incidence of any-grade hepatic enzyme disturbance with ipilimumab 3 mg/kg monotherapy is <4% and up to 15% when dosed at 10 mg/kg [24, 47]. Incidence of hepatitis in patients treated with anti-PD-1 ICIs is approximately 5%, but this rises to 30% in patients treated with combination ipilimumab and nivolumab [13, 28].

Acute pancreatitis has been reported but is rare [42]; asymptomatic elevation of lipase and amylase are more common. The role of the gut microbiome in determining treatment response and risk of toxicities, including colitis, in patients treated with ICIs is an area of active investigation.

### Diagnostic evaluation

In the setting of acute diarrhea, initial evaluation should exclude an infectious etiology (consider stool culture, *Clostridium difficile*, cytomegalovirus (CMV) DNA polymerase chain reaction (PCR), stool ova and parasites). Inflammatory markers (fecal leukocytes/lactoferrin, fecal calprotectin) and fecal occult blood test (FOBT) may help indicate whether there is an inflammatory process underlying the diarrhea. Screening tests for tuberculosis, human immunodeficiency virus (HIV) and hepatitis A and B should be considered if there is potential for use of systemic immunosuppression e.g. infliximab in the near future. Based on the IBD literature, risk of hepatitis C exacerbation is minimal; as a result, testing for hepatitis C is not recommended [48, 49].

#### Colitis

Radiologically, two distinct patterns of anti-CTLA-4-associated colitis have been observed on computed tomography (CT) imaging: a more common diffuse colitis characterized by mesenteric vessel engorgement, and a segmental colitis with moderate wall thickening and associated pericolonic fat stranding in a segment of pre-existing diverticulosis [50]. A fluorodeoxyglucose positron emission tomography (FDG-PET)/CT study can also demonstrate new FDG-avid diffuse colonic wall thickening in patients with immune-related colitis [50]. Colonoscopy is the most accurate means of evaluating the extent and severity of colitis and is recommended in appropriate cases since recent data suggest that the presence of ulceration on endoscopy predicts steroid-refractory disease [51]. For grade ≥ 2 diarrhea, systemic immunosuppression should be initiated promptly after ruling out infectious etiology. Colonoscopy can be considered if deemed clinically necessary, although it is worth noting that certain types of colitis may have a normal endoscopic appearance, with significant inflammatory features on histology. Therefore, routine mucosal biopsies should be performed for histological examination. In addition, pathology with immunohistochemical staining to rule out CMV infection is critical.

Histologically, colitis that follows treatment with anti-CTLA-4 antibodies is characterized by neutrophilic inflammation with increased intraepithelial lymphocytes, crypt epithelial cell apoptosis and few or no features of chronicity. Similarly, anti-PD-1-related colitis typically follows one of two patterns: active colitis with apoptosis (active inflammation, neutrophilic crypt micro-abscesses, increased crypt epithelial cell apoptosis, and presence of crypt atrophy/dropout) or lymphocytic colitis (increased intraepithelial lymphocytes in surface epithelium, surface epithelial injury, and expansion of the lamina propria). Pathological changes may also be visible outside the colon in the duodenum, stomach and/or small bowel [52].

#### Hepatitis

Liver function testing prior to initiation of ICIs, and again before each cycle of treatment, can help determine patterns of liver enzyme disturbance. Hepatitis following ICI therapy is typically detected on routine serum liver function tests. Other causes of liver damage such as viral infection, alcohol, other medications or cancer progression should be excluded. Other thromboembolic and outflow obstructive etiology should also be excluded through imaging. On radiologic evaluation, ipilimumab-associated hepatitis has been shown to present with non-specific and variable findings according to clinical severity [53]. Hepatomegaly, edema and enlarged lymph nodes in the periportal region, and attenuated liver parenchyma may be evident on CT and MRI. Liver biopsy, only necessary in complicated cases, may reveal predominantly hepatocyte injury (acute hepatitis pattern) with sinusoidal histiocytic infiltrates, central hepatic vein damage and endothelial inflammation similar to autoimmune hepatitis, or predominant bile duct injury (biliary pattern, with portal inflammation) [53, 54]; rarely, fibrin ring granulomas have also been reported [55].

### When to refer

If infectious work-up is negative, diarrhea due to previous immunotherapy exposure should be considered a possible etiology since colitis can wax and wane after an initial episode. Endoscopy and histology may provide further clarification, and the patient should be referred promptly to a gastroenterologist who is experienced managing patients with gastrointestinal adverse events after immunotherapy. There are reports about progression of colitis to chronic IBD long term [56] and such patients should be followed by a gastroenterologist long term.

## Endocrine adverse events

### Clinical presentation and epidemiology

The two most common endocrine irAEs are acute hypophysitis resulting in hypopituitarism (central hypothyroidism, central adrenal insufficiency, hypogonadotropic hypogonadism), and thyroid disease or abnormalities in thyroid function tests (primary hypothyroidism and thyroiditis). Other endocrinopathies such as primary adrenal insufficiency, T1DM, hypercalcemia, and hypoparathyroidism have been reported but are rare. The prevalence of these disorders varies greatly. This may be due to the non-specific presenting signs and symptoms, such as, headache, fatigue, anorexia and nausea, coupled with the fact that hormonal abnormalities are not uncommon in patients with advanced cancer. Diagnosis is also complicated by the fact that baseline screening for endocrine abnormalities is not routinely performed (other than thyroid function tests, in some cases), and corticosteroids may be initiated empirically for suspected irAEs, which interferes with subsequent endocrine testing. A low threshold of clinical suspicion is therefore warranted and, in the absence of alternate etiologies, a diagnostic work-up for endocrine dysfunction should be initiated.

### Diagnostic evaluation

Routine monitoring for clinical signs and symptoms of endocrinopathies, and patient education, are recommended. All patients should be tested before starting treatment for thyroid (thyroid-stimulating hormone [TSH] and free thyroxine [freeT4]), early morning adrenal (adrenocorticotropic hormone [ACTH] and cortisol) function, and glycemic control (glucose and glycated hemoglobin [HbA1c]). In situations where new elevation in glucose is noted, testing for blood or urinary ketones should be considered. Before each cycle, thyroid testing TSH and free T4) should be repeated, along with a baseline metabolic panel to allow monitoring of glycemic trends.. Routine monitoring with early morning ACTH and cortisol levels should be considered (every month for 6 months, then every 3 months for 6 months then every 6 months for 1 year).

#### Hypophysitis

Hypophysitis is most commonly seen with anti CTLA-4 antibody monotherapy (ipilimumab, with an incidence of ≤10% at a dose of 3 mg/kg and up to 17% at 10 mg/kg), and with combination ipilimumab/nivolumab (incidence ≤13%) [10, 13, 16, 17, 57]. The median time from starting ipilimumab to diagnosis of hypophysitis is 8–9 weeks, or after the third dose of ipilimumab [15, 58]. Symptoms commonly include headache (85%) and fatigue (66%); visual changes are uncommon. Clinical suspicion of hypophysitis is frequently raised when routine thyroid function testing shows a low TSH with low free T4, suggestive of a central etiology. Patients have various degrees of anterior pituitary hormonal deficiency, with central hypothyroidism being most commonly seen (>90%), followed by central adrenal insufficiency, which is also found in the majority of patients [59–61]. Both central hypothyroidism and adrenal insufficiency occur in >75% of patients and approximately 50% of patients present with panhypopituitarism (adrenal insufficiency plus hypothyroidism plus hypogonadism) [61–63]. On magnetic resonance imaging (MRI) of the sella, pituitary enlargement can precede the development of clinical and biochemical evidence of disease. MRI abnormalities, such as stalk thickening, suprasellar convexity, heterogeneous enhancement, and increased height of the gland as compared with baseline scans (when available) are present in most patients at the time of diagnosis. Resolution of pituitary enlargement is common, with all cases resolved on follow up scans after two months [60, 64].

All patients with suspected hypophysitis based on clinical findings (headache, fatigue) or biochemical evaluation (routine thyroid function testing showing low free T4 with low/normal TSH) should undergo further testing for diagnostic confirmation. Recommended tests, preferably conducted in the morning around 8 am, include thyroid function (TSH, free T4), adrenal function (ACTH, cortisol or 1 mcg cosyntropin stimulation test), gonadal hormones (testosterone in men, estradiol in women), follicle-stimulating hormone [FSH], luteinizing hormone [LH]) and MRI of the sella, with pituitary cuts. This should be done prior to administration of steroids. Strict criteria for diagnostic confirmation of hypophysitis are not currently available. Proposed confirmation criteria include ≥1 pituitary hormone deficiency (TSH or ACTH deficiency required) combined with an MRI abnormality, or ≥2 pituitary hormone deficiencies (TSH or ACTH deficiency required) in the presence of headache and other symptoms.

Management of confirmed hypophysitis includes replacement of deficient hormones (physiologic doses of steroids and thyroid hormone). In the presence of both adrenal insufficiency and hypothyroidism, steroids should always be started prior to thyroid hormone in order to avoid an adrenal crisis. High doses of steroids are necessary in the setting of severe headaches, vision changes or adrenal crisis. Both adrenal insufficiency and hypothyroidism appear to represent long term sequelae of hypophysitis and lifelong hormonal replacement is needed in most cases [59, 64–66]. All patients with adrenal insufficiency should be instructed to obtain and carry a medical alert bracelet.

#### Thyroid dysfunction

Thyroid dysfunction (hypothyroidism, hyperthyroidism, and thyroiditis) was reported in 6–20% of patients in large phase 3 clinical trials.

#### Hypothyroidism

Patients with unexplained fatigue, weight gain, hair loss, cold intolerance, constipation, depression and other recognized symptoms should be suspected of having hypothyroidism. Lab tests showing high TSH and low free T4 are indicative of biochemical hypothyroidism and, if present, additional testing for thyroid antibodies such as thyroid peroxidase (TPO) antibody is warranted. Patients with confirmed hypothyroidism should be started on thyroid hormone, with repeat TSH and free T4 levels evaluated 6–8 weeks later. Once a maintenance dose is identified (TSH within normal range) clinical and biochemical re-evaluation should be undertaken every 12 months.

#### Thyrotoxicosis

Thyrotoxicosis (high free T4 or total T3 with low or normal TSH) may occur secondary to thyroiditis or Graves’ disease. Thyroiditis is the most frequent cause of thyrotoxicosis and is seen more commonly with anti-PD1/PD-L1 drugs than with anti-CTLA-4 agents; Graves’ disease is very rare and occurs more commonly with anti-CTLA-4 drugs. Thyrotoxicosis due to thyroiditis may present with weight loss, palpitations, heat intolerance, tremors, anxiety, diarrhea and other symptoms of hypermetabolic activity, although these symptoms may be masked if the patient is taking beta-blockers. Most commonly, patients are asymptomatic (painless thyroiditis) and routine laboratory monitoring shows high free T4 or triiodothyronine (T3) levels, with low/normal TSH. A thyrotoxic phase occurs an average of one month after starting the drug. Additional tests can be undertaken when thyroiditis is suspected, primarily to rule out other causes of thyrotoxicosis such as Graves’ disease. These include thyroid stimulating hormone receptor antibody [TRAb] or thyroid stimulating immunoglobulin (TSI) and TPO as well as images when feasible: radioactive iodine uptake scan (RAIUS) or Technetium (Tc)-99 m [pertechnetate] thyroid scan if recent iodinated contrast was used. Thyroiditis is a self-limiting process and leads to permanent hypothyroidism after an average of 1 month after the thyrotoxic phase and 2 months from initiation of immunotherapy. Conservative management during the thyrotoxic phase of thyroiditis is sufficient. Non-selective beta blockers, preferably with alpha receptor-blocking capacity, may be needed in symptomatic patients. Repeat thyroid hormone levels should be performed every 2–3 weeks and thyroid hormone replacement initiated at the time of hypothyroidism diagnosis [59, 64].

#### Type 1 diabetes mellitus

Development of polyuria, polydipsia, weight loss, nausea and/or vomiting should prompt investigation for possible development or worsening of T1DM. Diagnosis and management of T1DM is based on recognized guidelines [67]. Tests for antibodies (glutamic acid decarboxylase [GAD65], anti-insulin, anti-islet cell A, zinc transporter 8 [Zn-T8]), C-peptide and insulin could distinguish between type 1 and type 2 disease.

### When to refer

An endocrinology consultation is recommended in all cases of suspected or confirmed hypophysitis, primary hypothyroidism, hyperthyroidism, thyroiditis, type 1 DM and all rare endocrinopathies.

## Pulmonary adverse events

### Clinical presentation and epidemiology

#### Pneumonitis

The most common lung toxicity observed in patients receiving ICI treatment is pneumonitis. The overall incidence of pneumonitis associated with PD-1/PDL-1 and CTLA-4-targeted therapies is <5%, with high-grade (≥grade 3) events occurring in 1–2% of patients. Higher rates have been reported for combinations of PD-1 and CTLA-4 inhibitors [68]. These numbers are not clinically trivial, as pneumonitis is one of the most common causes of ICI-related death. Moreover, the incidence of pneumonitis is increasing as therapeutic indications for ICIs expand, and more complex regimens are developed. Pneumonitis may present on imaging studies as cryptogenic organizing pneumonia (COP), nonspecific interstitial pneumonitis (NSIP), hypersensitivity pneumonitis (HP), or usual interstitial pneumonitis (UIP)/pulmonary fibrosis (PF). Clinical and radiographic findings of ICI-related pneumonitis may closely mimic pneumonia, lymphangitic spread of disease, cancer progression, and diffuse alveolar hemorrhage. The radiographic appearance of pneumonitis may be clinically asymptomatic or, alternatively, associated with new or worsening shortness of breath, cough, wheezing, chest pain, reduced exercise tolerance, fatigue with activities of daily living (ADL) and new or increasing requirement for supplementary oxygen. Acuity of onset and severity may also vary, suggesting the importance of vigilance and rapid response in some cases. Studies have suggested a higher incidence of any grade (3.6% vs. 1.3%) and severe (1.1% vs. 0.4%) pneumonitis with PD-1 inhibitors compared with PD-L1 inhibitors [69]. Combination therapies with anti-CTLA-4/anti-PD-1/PD-L1 immunotherapy and with ICI/cytotoxic combinations also confer a higher risk of pneumonitis versus ICI monotherapy [68, 70]. Higher rates of pneumonitis have also been reported among ICI-treated patients with non-small cell lung cancer (NSCLC) compared to patients with melanoma [71]. Pneumonitis onset appears earlier in cases of NSCLC (median [range]: 2.1 [0.2–27.4] months) versus melanoma (median [range]: 5.2 [0.2–18.1] months) [72]. IrAEs associated with other organ systems, including hepatitis, colitis, duodenitis, esophagitis, thyroiditis, hypophysitis, arthritis, myositis, vitiligo, nephritis, and anemia may occur in up to 50% of patients and confound therapy. These irAEs may occur concomitantly, precede or follow the development of pneumonitis. In patients with preexisting lung diseases, such as chronic obstructive pulmonary disease (COPD) or PF, the diagnosis of pneumonitis is particularly challenging and failure to recognize and treat pneumonitis in a timely manner could lead to poor clinical outcomes.

In addition to pneumonitis, ICI therapy has been associated with pleural effusions, pulmonary sarcoidosis and sarcoid-like granulomatous reactions. Sarcoid-like reactions have been reported following both CTLA-4 and PD-1/PD-L1-targeted therapies. Increased numbers of T helper 17 (Th17.1) cells are seen in the bronchoalveolar lavage (BAL) fluid of these patients, suggesting that TH17 cells may play an important role in the pathogenesis of this disease [73]. Sarcoidosis may be asymptomatic or present with cough, wheezing, fatigue and/or chest pain. Data in this area are scant at present, although case reports suggest that the development of sarcoidosis may be associated with prolonged cancer response [74, 75].

Treatment strategies for ICI related pneumonitis, based on pneumonitis grade, are detailed in Table 3. Patients with grades 1–2 pneumonitis may be managed as outpatients while those with pneumonitis grade 3 or higher typically require hospitalization. Drug withdrawal is the mainstay of treatment for pneumonitis of all grades. For patients with grade 1 pneumonitis, re-challenge following resolution of infiltrates and close follow-up is reasonable. In these patients, symptoms should be monitored every 2–3 days. A repeat chest CT should be performed prior to the next scheduled dose of ICI and if the infiltrates have resolved, ICI therapy may be cautiously resumed with close follow-up. Bronchoscopy should be considered for evidence of new or persistent infiltrates. Patients with grade 2 or higher pneumonitis may require oral/intravenous corticosteroids. Recrudescence of pneumonitis signs and symptoms has been reported following rapid steroid taper; a minimum 4–6 week taper is therefore recommended. Additional immunosuppression with infliximab and/or cyclophosphamide is warranted among patients with recalcitrant disease.

#### Sarcoidosis

Once a diagnosis of sarcoidosis is established, immunotherapy should be withheld, particularly in patients with extensive disease (stage ≥2), extrapulmonary disease involving critical organ systems (ocular, myocardial, neurologic, renal), or sarcoid-related hypercalcemia. Treatment for irAE-related sarcoidosis should be considered if there is 1) progressive radiographic change; 2) persistent and/or troublesome pulmonary symptoms; 3) lung function deterioration (total lung capacity (TLC) decline of ≥10%, forced vital capacity (FVC) decline of ≥15%; diffusing capacity of the lungs for carbon monoxide (DLCO) decline of ≥20%); 4) concomitant involvement of critical extrapulmonary organ systems; or 5) sarcoid-related hypercalcemia. These guidelines are extrapolated from standard management guidelines for sarcoidosis in the general population. Further investigations of sarcoidosis management in the ICI setting are needed.

### Diagnostic evaluation

#### Pneumonitis

The diagnosis of pneumonitis is suggested by the presence of new or progressive pulmonary infiltrates and ground glass changes on lung imaging studies. The infiltrates are typically bilateral, but may be asymmetric. CT imaging is more reliable than chest radiographs in identifying these changes, and is the imaging modality of choice. Baseline and ongoing oxygen saturation (at rest and on ambulation) should be monitored in all patients, as well as chest CT, pulmonary function tests (PFTs), and a 6-min walk test (6MWT). A pulmonology consult is warranted in any patient with suspected pneumonitis. Atypical symptoms such as fever and productive cough should also trigger an infectious disease consultation. Fiberoptic bronchoscopy with BAL may be helpful in excluding competing diagnoses. Lung biopsies are typically not warranted, but may be useful in the setting of suspicious lesions and unexplained lymphadenopathy.

#### Sarcoidosis

The diagnosis of pulmonary sarcoidosis is suggested by radiographic evidence of intrathoracic lymphadenopathy and irregular densities, coupled with histologic evidence of epithelioid non-caseating granulomas obtained from endobronchial ultrasound (EBUS), fine needle aspiration (FNA) or transbronchial lung biopsy (TBBx). Since sarcoidosis can mimic malignant disease progression, both clinicians and radiologists should be aware of this possibility. Confirmation requires exclusion of infections and other competing diagnoses. Patients may also present with extrapulmonary manifestations of sarcoidosis. Therefore, once the diagnosis is established an eye examination and baseline electrocardiogram should be considered to investigate involvement of other organ systems. The natural history of irAE-related sarcoidosis is not known and treatment strategies for sarcoid in this setting have not been established.

### When to refer

Referral to a pulmonary specialist for bronchoscopy should be pursued in all patients with radiographic and/or clinical evidence of pneumonitis. Such evidence includes new pulmonary infiltrates on lung imaging, or new or worsened hypoxemia, dyspnea or cough. Unexplained lymphadenopathy or atypical pulmonary nodules and densities should also prompt a pulmonary referral. Infectious disease consultation should be considered for patients with ≥ grade 2 pneumonitis. Long-term specialist follow-up is also advisable in any patient with confirmed immune-related lung disease.

## Rheumatologic/musculoskeletal adverse events

### Clinical presentation and epidemiology

Recognizing rheumatologic and musculoskeletal irAEs in the oncology setting is challenging due to the broad range of potential presenting symptoms and the prevalence of musculoskeletal complaints in the general population. Although a paucity of epidemiological data limits our understanding of the true incidence of these irAEs, they are increasingly reported across care settings. Since delayed diagnosis and treatment can lead to long-term disability, and disorders may become chronic and require ongoing immunosuppressive/immunomodulatory therapy, it is important to understand typical symptom presentation and recommended management. Preserving quality of life and ability to perform ADL is a priority.

One of the most commonly reported rheumatologic irAEs is an inflammatory oligo or polyarthritis that can lead to rapid joint damage and may persist after discontinuation of immunotherapy. Arthralgia has been reported in approximately 15% of patients receiving ICIs, but the incidence of inflammatory arthritis, which is typically grade 2 or less, has not yet been systematically reported [76]. Arthritis is rarely the sole irAE, with most patients having other organ systems involvement. In a small series, the median time to onset was five months after starting ICI therapy. Clinically, three phenotypes have been described: 1) predominantly large joint reactive arthritis that, on occasion, develops in association with conjunctivitis and uveitis; 2) polyarthritis resembling rheumatoid-like arthritis, affecting the small joints of the hand (metacarpophalangeal [MCP], proximal interphalangeal [PIP] joints or wrist), rarely associated with typical rheumatoid factor (RF) or anti-citrullinated protein antibodies (ACPA), but potentially erosive; and 3) seronegative, oligo and polyarthritis, typically starting in the medium/large joints, characterized by synovitis and involvement of tendons and entheses, with or without joint erosions. Combination anti-CTLA-4/anti-PD-1 therapy is associated with a greater risk of arthritis than monotherapy, although incidence is unaffected by drug or type of malignancy. Management often requires moderate-dose corticosteroids, sometimes in conjunction with steroid-sparing immunomodulators and disease-modifying anti-rheumatic drugs (DMARDs) including tumor necrosis factor inhibitors (TNFi), methotrexate, leflunomide, sulfasalazine, and hydroxychloroquine. Persistence of inflammatory arthritis up to two years after discontinuation of ICIs has been seen, with ongoing requirement for immunomodulatory therapy. Beyond arthritis, less commonly reported rheumatologic irAEs recognized in the context of ICI therapy include sicca, with severe eye and mouth dryness, and parotitis; inflammatory myositis, most commonly resembling polymyositis, occasionally resulting in rhabdomyolysis; vasculitides including giant cell arteritis (GCA) and polymyalgia rheumatica (PMR); systemic lupus erythematosus (SLE) and sarcoidosis [76, 77].

One of the primary difficulties in ensuring accurate reporting of rheumatologic irAEs is the nature of severity grading in the current CTCAE. The current version (version 4) classifies many clinically significant rheumatologic events that require corticosteroids or immunomodulatory treatment as grade 1/2, whereas the rheumatology CTCAE (rCTCAE) compiled by the Outcome Measures in Rheumatology network (formerly Outcome Measures in Rheumatoid Arthritis Clinical Trials; OMERACT) (RCTCAE version 2.0) [34] classifies similar symptoms one or two severity grades higher. Of particular importance, the current CTCAE classifies impairment in instrumental ADLs (taking medications, preparing meals, housework, using transportation) as grade 2, despite the fact that this represents a considerable degree of functional disability and loss of independence. This also has implications for the detection of “clinically significant” musculoskeletal irAEs in clinical trial databases.

Current CTCAE terms for musculoskeletal symptoms (e.g. arthritis and myositis) are not easily converted to clinically relevant descriptors. Lack of precision may result in diffusion of an irAE signal, distorting the epidemiological landscape. For example, oncologists must choose between several different codes to document a swollen joint (joint effusion, joint pain, joint function, arthritis) or muscle weakness (myalgia, muscle weakness, change in lower extremity function). As such, it may be more appropriate to aggregate similar coding subtypes to better reflect the true incidence of musculoskeletal irAEs.

### Diagnostic evaluation

A diagnostic algorithm for inflammatory arthritis has recently been reported [78]. The SITC Toxicity Management Working Group evaluated and discussed this algorithm and made suggestions for its modification. The revisions are noted below.

Grade 1: Joint examination (swelling/tenderness), functional assessment, consider rheumatology referral, especially if symptoms persist.

Grade 2/3: Joint examination, functional assessment, consider laboratory testing (antinuclear antibody [ANA] rheumatoid factor [RF], cyclic citrullinated peptide antibody [anti-CCP], erythrocyte sedimentation rate [ESR]/ C-reactive protein [CRP]). Consider imaging (plain X-ray of affected joints, joint MRI and/or musculoskeletal ultrasound).

### When to refer

All patients with CTCAE ≥ grade 2 inflammatory arthritis should be referred to rheumatology. Also consider referring any patient whose symptoms persist for >6 weeks or who requires >20 mg prednisone (or equivalent) daily that cannot be tapered to <10 mg/day within 4 weeks [78]. All patients with suspected myositis, presenting with muscle weakness and elevated creatine kinase (CK), should be referred to rheumatology or neurology, as this can be a life-threatening adverse event.

Because erosive, irreversible joint damage has been seen within weeks of symptom onset, early involvement of rheumatologists is recommended to determine if additional disease-modifying therapy beyond steroids is required.

For other suspected rheumatologic manifestations (e.g. vasculitis, myositis, scleroderma, etc.), rheumatology referral is advisable even if the symptoms are mild, to ensure that appropriate diagnostic testing and optimal management can be coordinated to prevent permanent organ damage.

## Infusion reactions

### Clinical presentation and epidemiology

Infusion reactions may present with constitutional symptoms such as fever, rigor, pruritus, hypotension, dyspnea, chest discomfort, rash, urticaria, angioedema, wheezing or tachycardia, as well as the possibility of anaphylaxis requiring urgent intervention. Infusion reactions (all grades) are reported in 25% of patients receiving avelumab (premedication with acetaminophen and an antihistamine is recommended) [7] and in less than 10% of patients receiving other approved immune checkpoints inhibitors [5–9, 79–81]. Infusions of ipilimumab appear to be well-tolerated, with a low incidence (<6%) of infusion reactions even when the infusion is delivered over 30 min (as opposed to the standard 90 min timeframe) when patients are pre-medicated with diphenhydramine and/or corticosteroids [82]. Severe/life-threatening infusion reactions occurred in less than 2% of the patients. Mild to moderate reactions are managed with symptomatic treatment and by reducing the rate or temporarily interrupting the infusion [4–9]. Severe/life-threatening reactions should be managed promptly and in accordance with the institutional guideline for infusion reactions; permanent discontinuation is recommended for such cases (grades 3 or 4) [80].

### Diagnostic evaluation

Infusion reactions are common to many cancer treatments and appropriate training and procedures should be in place while patients are receiving an immunotherapy infusion. The severity of an infusion reaction should be rapidly assessed and appropriate treatment implemented in accordance with the institutional guideline. Life-threatening reactions with hypoxia and/or shock should be aggressively managed [80].

### When to refer

Cancer patients often receive more than one drug during infusion; patients with severe or life-threatening reactions (CTCAE grade 3 or 4) should therefore be referred to an allergist. Appropriate assessment and counseling could prevent future re-exposure to drugs that have previously caused severe reactions.

## Uncommon immune-related adverse events

### Cardiovascular adverse events

#### Clinical presentation and epidemiology

Cardiac irAEs due to ICIs may present with non-specific symptoms such as fatigue and weakness. However, more typical cardiac symptoms of chest pain, shortness of breath, pulmonary or lower extremity edema, palpitations, irregular heartbeat, rapid onset of heart failure symptoms or new heart block on electrocardiogram (ECG) can occur at any time, more frequently within the first few months of treatment. Other signs and symptoms may include muscle pain or syncope. Patients who develop immune toxicities of other organ systems may also develop cardiovascular toxicities, potentially with symptoms that overlap with myositis (myalgias, rhabdomyolysis) or myocarditis or pericarditis (fever, chest pain with inspiration, diffuse ST elevation on ECG), making accurate diagnosis a considerable challenge. It is suggested that there may be a link between rhabdomyolysis/myositis, vasculitis and cardiac toxicity. However, myocarditis, pericarditis and cardiac dysfunction due to ICIs are rare and the true incidence is unknown; current estimates suggest less than 1% of patients [22]. Moreover, due to varying definitions of cardiotoxicity [83], the obscurity of CTCAE entries for some cardiac irAEs, especially myocarditis, and the absence of systematic monitoring or coding mechanism for cardiac events in immunotherapy trials, cardiac irAEs are likely under-reported. In particular, myocarditis is a difficult diagnosis to make in any clinical situation, but especially in a patient being actively treated for cancer [84]. The expert consensus is to have high vigilance for development of cardiac symptoms in all patients, but especially in those with evidence of myocarditis, vasculitis or myositis.

Cardiac irAEs are seen across the ICI drug class, with higher incidence in patients taking combination anti-CTLA-4/anti-PD-1 treatment compared to monotherapy. Patients, including those with known cardiac comorbidities, should not be denied therapy with ICIs solely on the basis of the potential for cardiotoxicity, but the level of vigilance has to be raised. The non-specific presentation of cardiac irAEs and potential to cause rapid clinical deterioration with a higher than acceptable rate of mortality with cardiac toxicity, make it imperative to maintain a low threshold for clinical suspicion and early specialist referral.

#### Diagnostic evaluation

At baseline, prior to initiating ICI therapy, it is suggested that a judicious combination of biomarkers (e.g., troponin I or T, brain natriuretic peptide [BNP] or N-terminal pro B-type natriuretic peptide [NT pro-BNP], total CK, fasting lipid profile, total CK and an electrocardiogram [ECG] be evaluated in all patients). Myocarditis is very rare but other potentially serious cardiac manifestations (life-threatening rhythm disturbances and acute coronary syndromes) are reported more commonly [85]. Since the major indicator of suspicion for both myocarditis and acute coronary syndrome is elevated troponin, a fasting lipid profile serves as an important screening tool to distinguish between atherosclerosis-related troponin elevation and potential myocarditis. Two-dimensional echocardiography (2-D Echo) may also be warranted in high-risk patients with cardiac history, symptoms of dyspnea, or if initial tests are abnormal. Serial ECGs and cardiac biomarker testing should be considered, particularly in patients with abnormal baseline investigations or suspicious symptoms. There are no current recommendations for the appropriate time interval between tests. Patients who develop concerning symptoms while undergoing ICI therapy should have chest imaging to exclude pulmonary embolism, pneumonitis, or pulmonary edema, as well as an ECG; cardiac biomarkers done at baseline evaluation should be retested. A repeat 2D Echo should be considered in any patient who has significant dyspnea or abnormal cardiac safety screening tests.

#### When to refer

An accurate baseline CV risk assessment should be undertaken, including consultation with a cardiologist if appropriate, in any patient who has multiple CV risk factors or established CV disease at the onset of immune therapy. Immediate referral is warranted for any patient who develops abnormal cardiac test results during the course of ICI therapy. Since myocarditis can rapidly lead to death, patients with suspected or documented myocarditis should be admitted to the hospital for cardiac monitoring. Patients with confirmed myocarditis should receive emergent intervention with high dose corticosteroids, and immediate discontinuation of immunotherapy. Until data are available (e.g., cut-off levels of troponin) to determine when to start corticosteroids in patients with possible (as opposed to confirmed) myocarditis, this decision should be made on a case by case basis. The importance of active, ongoing consultation with a cardiologist to discuss the risk/benefit of continuing ICI therapy, starting steroids, or instituting other cardiac treatments, cannot be overstated.

### Hematologic adverse events

#### Clinical presentation and epidemiology

Although rare, hematologic irAEs have been described following ICI treatment and the literature includes case reports of hemolytic anemia, red cell aplasia, neutropenia, thrombocytopenia, myelodysplasia and hemophilia A [15, 28, 86]. An active hematologic irAE also needs to be distinguished from transient changes in laboratory values that can occur during initiation of an immune response. Post treatment lymphcytosis, eosinophilia, neutrophilia and monocytosis can be observed and are not typically clinically significant though some reports suggest they may be prognostic [87]. Persistent post treatment cytopenias or progressive cytopenias should be evaluated for autoimmune causes as well as with a peripheral smear, reticulocyte count and assessment for hemolysis [88]. Causal attribution is complicated by the fact that malignant disease and its complications can also lead to cytopenias. Since the CTCAE definition of thrombocytopenia describes absolute platelet levels rather than an indication of changes in cell number, it is not a reliable tool for evaluating potentially life-threatening ICI-induced thrombocytopenia.

#### Diagnostic evaluation

Complete blood count (CBC) should be monitored at the start of immune therapy, at intervals during treatment, and periodically in long-term survivors who are no longer receiving treatment. Development of anemia should prompt evaluation for common causes such as GI bleeding, cancer-related anemia or cancer progression, or causative drugs, including a work up for hemolysis. If the source of anemia cannot be identified, bone marrow biopsy may be indicated to rule out red cell aplasia. Similarly, any patient who develops thrombocytopenia or neutropenia should be evaluated for potential causes including medication-related cell destruction or disease progression; in cases where an obvious cause cannot be identified, an autoimmune cause should be considered and investigated accordingly.

#### When to refer

In general, patients with unexplained cytopenias should be referred to hematology for evaluation.

## Renal adverse events

### Clinical presentation and epidemiology

Overall, renal irAEs are considered rare, occurring in 2% (ICI monotherapy) to 5% (combination ipilimumab/nivolumab) of patients taking ICIs, with underlying pathology only beginning to be characterized and reported [89, 90]. Most reports document isolated cases of interstitial nephritis with specific agents and regimens, such as anti-PD-1 monotherapy, and combination anti-CTLA-4/PD-1 treatment, in melanoma [91, 92]. Nephritis has not been associated with anti-PD-L1 monotherapy to date. Three cases of acute renal failure were also reported during a study of nivolumab and doublet platinum chemotherapy in NSCLC [93]. There are also case reports of lupus nephritis [94] and granulomatous nephritis [95, 96] following ipilimumab treatment, and a single case of nephritis described after treatment with avelumab [97]. However, recent data suggest the incidence of renal irAEs may be under-reported with low-grade kidney injury affecting 25–29% of patients taking certain ICIs [90]. The onset of renal injury seen with PD-1 inhibitors usually occurs 3–10 months after initiation of treatment, whereas irAEs secondary to anti-CTLA-4 agents tend to have an earlier onset, after 2–3 months [90]. Renal toxicity from ICIs is usually asymptomatic, although oliguria, hematuria, peripheral edema and anorexia are occasionally reported. Management requires considerable clinical judgment.

### Diagnostic evaluation

Diagnosis of renal impairment may be complicated by concomitant medications that precede, or are prescribed during the course of, immunotherapy treatment. Nonetheless, evidence of gradually rising serum creatinine should prompt clinical suspicion. As such, serum creatinine should be monitored on starting immunotherapy treatment, and at intervals throughout the treatment course. If creatinine remains elevated for >2–3 days, monitor weekly (grade 1) or every 2–3 days (grade 2). It is important to exclude other causes of renal dysfunction through active inquiry about new medications, correction of dehydration and, possibly, additional investigations such as bladder and/or renal ultrasound, urinalysis, assessment of serum electrolytes, or other studies based on history. In suspected cases of immune-related renal disease, renal biopsy should be considered to confirm etiology and guide management. Because renal toxicity typically resolves, treatment can resume if grade 2–3 adverse events resolve promptly, but therapy should be discontinued in the face of persistent or recurrent grade 2–3 adverse events, or emergence of grade 4 toxicity. A nephrology consult should be considered for any persistent ≥ grade 3 renal impairment, or for recurrent renal toxicity following a corticosteroid trial.

### When to refer

A nephrology consult should be considered in patients with persistent grade 2–3 elevation in creatinine, ≥ 3-fold increase in creatinine over baseline, or whenever there is evidence of metabolic change consistent with renal failure.

## Neurologic adverse events

### Clinical presentation and epidemiology

Neurologic irAEs are uncommon, with an overall incidence of <4% following treatment with anti-CTLA-4 antibodies, 6% with anti-PD-1 antibodies, and 12% with combination therapy involving both [98]. Most events are mild and present with non-specific symptoms such as headache; irAEs grade 3 or higher occur in <1% of patients [98]. Examples of neurologic irAEs include autoimmune encephalitis, myasthenic syndrome/myasthenia gravis, Guillain-Barré syndrome, peripheral sensorimotor neuropathies, Posterior Reversible Encephalopathy Syndrome (PRES), aseptic meningitis and transverse myelitis [99]. Relevant CTCAE terms include encephalopathy, leukoencephalopathy, peripheral motor neuropathy, peripheral sensory neuropathy reversible posterior leukoencephalopathy syndrome, and ‘nervous system not otherwise specified’. Common presenting features of autoimmune encephalitis, meningitis and encephalopathy include altered mental status, headache, seizures, focal neurologic abnormalities and PRES.

### Diagnostic evaluation

Diagnostic work-up should include history and physical examination with full neurologic exam in all patients. Evaluation of possible autoimmune encephalitis, meningitis and encephalopathy should include lumbar puncture and brain MRI, with and without contrast; it is important to rule out infection, screen for unsuspected central nervous system (CNS) metastasis and/or leptomeningeal spread. Paraneoplastic syndromes should also be considered. Diagnostic evaluation of suspected peripheral sensorimotor neuropathies should include differential diagnosis of disorders including, but not limited to, diabetic neuropathy and vitamin B12 deficiency. Consider imaging as appropriate, as well as nerve biopsy; this is a diagnosis of exclusion, but in most cases it is a clinical diagnosis.

### When to refer

Neurology consultation is recommended for all neurologic irAEs grade 2 and higher.

## Ophthalmologic adverse events

### Clinical presentation and epidemiology

Ocular irAEs, predominantly uveitis (anterior more commonly than posterior or panuveitis) are reported in <1% of patients taking ICIs [13, 15]. There have also been reports of orbital inflammation, episcleritis, blepharitis, optic nerve swelling, peripheral ulcerative keratitis and Vogt-Koyanagi-Harada picture with localized serous retinal detachment [100–102]. Patients prescribed ICIs should be advised to alert the clinician to new onset of blurred vision, floaters, flashing lights, changes in color vision, eye redness, photophobia or light sensitivity, visual distortion and visual field changes, scotomas, tender eyes or pain on eye movement, eyelid swelling or proptosis or double vision. Patient counseling is crucial to ensure that early signs and symptoms are recognized in a timely manner.

### Diagnostic evaluation

Although prompt ophthalmologic referral is important in ALL cases of visual complaints, certain tests can be performed by the oncologist in the office. These include examination for visual acuity, which can be done using an eye chart on a smart phone with the patient wearing reading glasses for near vision or glasses for distant vision, as necessary; color vision; red reflex; pupils (equal, round, reactive), including testing for an afferent pupillary defect, which can indicate optic nerve or extensive retinal disease; and penlight inspection of the anterior part of the eye. Direct ophthalmoscopy to examine the optic nerve and retina is unlikely to be useful for diagnosis of retinal or optic nerve issues when performed by a non-ophthalmology-trained physician. Ocular irAEs may be asymmetric so it is important to examine each eye separately. Ocular irAEs are frequently accompanied by irAEs in other systems, especially colitis, so broader systems inquiry is helpful.

### When to refer

Complaints of red, painful, dry or irritated eyes, or visual disturbance in a patient taking an ICI should alert the clinician to the need for immediate ophthalmological referral for diagnosis, classification and management, which can be difficult for the oncologist since different ocular pathologies and grades may present with similar symptoms and detailed ophthalmological evaluation needs to be performed by an ophthalmologist, including a slit lamp exam and dilated fundus exam. Sometimes grade 2 or 3 severity irAEs may only present with asymptomatic or mild changes in vision, and time to ophthalmology access can vary depending on the setting (academic versus community hospital). Clinical suspicion and prompt referral are therefore essential. Starting systemic or topical treatment with corticosteroids prior to conducting an eye exam should be avoided unless systemic steroids are indicated for a concurrent, non-ophthalmological toxicity, since it may worsen ocular conditions that are due to infection (e.g., herpetic keratitis/uveitis) or may mask accurate diagnosis and severity grading when the patient is examined by an ophthalmologist. Urgent referral is warranted for any grade 3 or 4 irAEs, but even patients with grade 1 or 2 toxicities should undergo full ophthalmological evaluation, proper grading, work up and treatment evaluation by an ophthalmologist within a few days. Puffy eyelids may indicate early preseptal cellulitis, which requires systemic antibiotic treatment. Warning signs (eyelid swelling with pain and erythema, proptosis, pain with eye movements, movement restriction/diplopia, vision changes) should prompt urgent ophthalmology referral.

## Conclusions

As the number of patients treated with checkpoint inhibitors grows, and the volume of real-world data increases, the etiology and characterization of immunotherapy-related toxicities will become clearer, and management more targeted and effective. Since adverse events may occur late, even after terminating active treatment, and there is a potential for long-term chronic complications, constant vigilance and early recognition and treatment of immune-related adverse events is important. Until prospective clinical data are available, the consensus recommendations provided here, on the diagnosis and management of immune checkpoint inhibitor-related adverse events, will hopefully serve as a starting point to help clinicians provide timely and effective management of immune-related toxicities in their patients with cancer.

## Additional file

Additional file 1: Table S1. Recommended management of uncommon dermatologic immune-related adverse events. (DOCX 24 kb)

## Abbreviations

2-D echo: Two-dimensional echocardiogram/echocardiography
6MWT: 6 min walk test
AACR: American Association for Cancer Research
ACCC: Association of Community Cancer Centers
ACPA: Anti-citrullinated protein antibodies
ACTH: Adrenocorticotropic hormone
ADCC: Antibody-dependent cell-mediated cytotoxicity
ADL: Activities of daily living
ALT: Alanine aminotransferase
ANA: Antinuclear antibody
Anti-CCP: Cyclic citrullinated peptide antibody
Anti-RF: Anti-rheumatoid factor (anti-RF)
Anti-TNF: Anti-tumor necrosis factor
ASCO: American Society of Clinical Oncology
AST: Aspartate aminotransferase
ATG: Anti-thymocyte globulin
BAL: Bronchoalveolar lavage
BID: Two times daily
BNP: B-type natriuretic peptide
BSA: Body surface area
CBC: Complete blood count
CK: Creatine kinase
CMP: Complete metabolic panel
CMV: Cytomegalovirus
CNS: Central Nervous System
COP: Cryptogenic organizing pneumonia
COPD: Chronic obstructive pulmonary disease
CRP: C-reactive protein
CT: Computed tomography
CTCAE: Common Terminology Criteria for Adverse Events
CTLA-4: Cytotoxic T lymphocyte-antigen-4
DIC: Disseminated intravascular coagulation
DLCO: Diffusing capacity of the lungs for carbon monoxide
DMARDS: Disease modifying anti-rheumatic drugs
DRESS: Drug rash with eosinophilia and systemic symptoms
EBUS: Endobronchial ultrasound
ECG: Electrocardiogram
ESR: Erythrocyte sedimentation rate
FDA: U.S. Food and Drug Administration
FDG-PET: Fluorodeoxyglucose positron emission tomography
FNA: Fine needle aspiration
FOBT: Fecal occult blood test
FreeT4: Free thyroxine
FSH: Follicle-stimulating hormone
FVC: Forced vital capacity
GAD65: Glutamic acid decarboxylase
GCA: Giant cell arteritis
GI: Gastrointestinal
HbA1c: Glycated hemoglobin
HBcAb: Hepatitis B core antibody
HBsAb: Hepatitis B surface antibody
HBsAg: Hepatitis B surface antigen
HCAb: Hepatitis C antibody
Hgb: Hemoglobin
HIV: Human immunodeficiency virus
HP: Hypersensitivity pneumonitis
HRT: Hormone Replacement Therapy
HSV: Herpes simplex virus
IBD: Inflammatory bowel disease
ICIs: Immune checkpoint inhibitors
ICU: Intensive care unit
IF: Immunofluorescence
IgE: Immunoglobulin E
IgG1: Immunoglobulin G1
IgG4: Immunoglobulin G4
irAEs: Immune-related adverse events
IVIG: Intravenous immunoglobulin
LH: Luteinizing hormone
LLN: Lower limit of Normal
mAbs: Monoclonal antibodies
MCP: Metacarpophalangeal
MedDRA: Medical Dictionary for Regulatory Activities
MRI: Magnetic resonance imaging
NCCN: National Comprehensive Cancer Network
NCI: National Cancer Institute
NIH: National Institutes of Health
NSCLC: Non-small cell lung carcinoma
NSIP: Nonspecific interstitial pneumonitis
NT pro-BNP: N-terminal pro B-type natriuretic peptide
ONS: Oncology Nursing Society
PASI: psoriasis area severity index
PCR: Polymerase chain reaction
PD-1: Programmed cell death protein-1
PD-L1: Programmed cell death-ligand 1
PF: Pulmonary fibrosis
PFTs: Pulmonary function tests
PIP: Proximal interphalangeal
PMR: Polymyalgia rheumatic
PRES: Posterior Reversible Encephalopathy Syndrome
QID: Four times daily
RA: Rheumatoid arthritis
RAIUS: Radioactive iodine uptake scan
SITC: Society for Immunotherapy of Cancer
SJS/TEN: Stevens-Johnson Syndrome/toxic epidermal necrolysis
SLE: Systemic lupus erythematosus
T1DM: Type I Diabetes
T3: Triiodothyronine
TBBx: Transbronchial lung biopsy
Tc: Technetium
Th17.1: T helper 17 cells
TLC: Total lung capacity
TNFi: Tumor necrosis factor inhibitor
TPO: Thyroid peroxidase
TRAb: Thyroid-stimulating hormone receptor antibody
TSH: Thyroid-stimulating hormone
TSI: Thyroid-stimulating immunoglobulin
UIP: Usual interstitial pneumonitis
ULN: Upper Limit of Normal
UVB: Short wave ultraviolet B
VZV: Varicella zoster virus
Zn-T8: Zinc transporter 8
