# Supplementary material for: Managing toxicities associated with immune checkpoint inhibitors: consensus recommendations from the Society for Immunotherapy of Cancer (SITC) Toxicity Management Working Group
Source: J Immunother Cancer. 2017 Nov 21;5:95. doi: 10.1186/s40425-017-0300-z (PMC5697162; doi:10.1186/s40425-017-0300-z)
Supplement: Additional file 1: Table S1. — Recommended management of uncommon dermatologic immune-related adverse events. (DOCX 24 kb) [file 40425_2017_300_MOESM1_ESM.docx]

| *Supplemental Table 1: Recommended management of uncommon dermatologic immune-related adverse events* | | |  |
| --- | --- | --- | --- |
| **DERMATOLOGIC** | | | **Specialist referral?** |
| Bullous dermatitis | | |  |
| Grade | **Description** | **Management** |  |
| 1 | Asymptomatic; blisters covering <1% BSA | Urgent referral for blisters ≥1%Must exclude SJS/TENNon-acute dermatology referral for diagnosis <1%Skin biopsy with direct immunofluorescence (IF), serum for indirect IF, CBC with differential, serum IgE level, PCR or viral cultures of blister base for herpes simplex virus (HSV)/varicella zoster virus (VZV)Topical corticosteroids while biopsy pending  - - Class I topical corticosteroid (clobetasol, halobetasol, betamethasone dipropionate) for body; class V/VI corticosteroid (aclometasone 0.05%, desonide 0.05%, hydrocortisone 2.5% cream) for face.  No dose modification | **✓** |
| 2 | Blisters covering 10 - 30% BSA; painful blisters; limiting instrumental ADL | Same-day dermatology referralSkin biopsy with direct IF, serum for indirect IF, CBC with differential, serum IgE, PCR or viral cultures of blister base for HSV/VZVTopical corticosteroids  - - Class I topical corticosteroid (clobetasol, halobetasol, betamethasone dipropionate) for body; class V/VI corticosteroid (aclometasone 0.05%, desonide 0.05%, hydrocortisone 2.5% cream) for face.  Oral antihistamine (hydroxyzine 25mg QID or equivalent)Consider oral corticosteroids if rapidly progressive  - - Prednisone 0.5 – 1mg/kg/day until rash resolves to ≤ grade 1  Consider doxycycline 100mg BID, nicotinamide 500mg BID | **✓** |
| 3 | Blisters covering >30% BSA; limiting self-care ADL | Same day referral to dermatology or admit with dermatology consultSkin biopsy with direct IF, serum for indirect IF, CBC with differential, IgESystemic corticosteroidsPrednisone 1mg/kg (or equivalent dose of methylprednisolone), tapering over two weeksAdd steroid-sparing agent if autoimmune bullous disorder is confirmed*See bullous pemphigoid recommendations | **✓** |
| 4 | Blisters covering >30% BSA; associated with fluid or electrolyte abnormalities; ICU care or burn unit indicated | Admit, dermatology consultSystemic corticosteroids per admitting teamPrednisone 1-2mg/kg (or equivalent dose of methylprednisolone)Steroid-sparing agent per consultant if autoimmune bullous disorder is confirmed | **✓** |
| Note: Empiric treatment with valacyclovir/acyclovir is recommended in any patient with clear evidence of dermatomal varicella zoster | | | |
| Bullous pemphigoid* | | |  |
| Grade | **Description** | **Management** |  |
| Not defined in CTCAE but comprises clear, fluid filled tense bullae and vesicles with significant itch. May involve the mucosa. | | - Refer for dermatology consult for diagnosis - 1st Line: Topical corticosteroids - 2nd Line: Oral corticosteroids (prednisone 0.5-1mg/kg, or equivalent dose of methylprednisolone, tapered over 2 weeks - 3rd Line: Minocycline/doxycycline 100mg BID with nicotinamide 500mg BID added when oral corticosteroids are initiated - 4th Line: Rituximab (375 mg/m2 once weekly for 4 weeks) or omalizumab (if IgE high)^§^ - 5^th^ Line: Methotrexate^§^, titrating up to 25mg weekly | **✓** |
| Notes: Empiric treatment with valacyclovir/acyclovir is recommended in any patient with clear evidence of dermatomal varicella zoster* Recommendations provided are based on case reports, series and expert consensus. Use of suggested therapies must be discussed with medical oncology based on individual patient considerations. The impact of these therapies on the anti-tumor immune response and efficacy of cancer treatment is unknown and requires further research | | | |
| Psoriasis/Psoriasisiform dermatitis* | | |  |
| Grade | **Description** | **Management** |  |
| 1 or 2 | Not defined in CTCAE but comprises pink to red papules (<1cm) and plaques with a white or silvery scale. May be associated with nail changes and inflammatory joint symptoms. | Non-acute dermatology referralTopical corticosteroids/tazarotene/calcipotriene | **✓** |
| 3 |  | Refer for dermatology consult1^st^ line: Topical corticosteroids and calcineurin inhibitors2^nd^ line: Phototherapy using narrow-band UVB per standard psoriasis protocols3^rd^ line: Acitretin, starting 10mg daily and titrating up to 25mg daily after 8 weeks if not sufficientIf joints involved, refer for rheumatology consult and consider the following:  - 4^rd^ line: Apremilast 30mg BID - 5^th^ line: Methotrexate^§^ - 6^th^ line: TNF inhibitor ^§^ | **✓** |
| Notes:Drug list does not indicate order of preference. Refer to CTCAE for grading system, which should not be confused with the PASI system.*Apremilast and tazarotene may be associated with delays in coverage authorization. Apremilast can also trigger diarrhea that could complicate diagnosis or interpretation of immune-related colitis.  1. Retrospective cohort studies show that in RA patients treated with TNF inhibitors there is no increase in solid cancers and no increased recurrence of breast cancer. More data are needed to determine long term lack of harm across malignancies and inhibitors.  *Recommendations provided are based on case reports, series and expert consensus. Use of suggested therapies must be discussed with medical oncology based on individual patient considerations. The impact of these therapies on the anti-tumor immune response and efficacy of cancer treatment is unknown and requires further research§ Risk/benefit must be reviewed carefully with medical oncology***IMPORTANT*: Joint involvement warrants a rheumatology consult** | | | |
| Lichenoid dermatitis* | | |  |
| Grade | **Description** | **Management** |  |
| Not defined in CTCAE but characterized by discrete, flat-topped pink or violaceous papules that may have some scale. Mucosa may be involved with erosions, ulcerations or Wickham’s striae. | | Non-acute dermatology referral  - 1st Line: Topical corticosteroids   - Class I topical corticosteroid (clobetasol, halobetasol, betamethasone dipropionate) for body; class V/VI corticosteroid (aclometasone 0.05%, desonide 0.05%, hydrocortisone 2.5% cream) for face.   - Dexamethasone swish and spit for oral involvement (referral to oral medicine for severe involvement) AND oral antihistamines (hydroxyzine 25mg QID or equivalent) - 2nd Line: Phototherapy using narrow-band UVB per standard protocol - 3rd Line: Acitretin 10mg daily and titrate up to 25mg daily after 8 weeks if not sufficient - 4th Line: Metronidazole 500mg BID for 3 month trial; hydroxychloroquine 200 or 400 mg daily (<6.5 mg/kg/day) - 5th Line: Methotrexate^§^ - 6th Line: Systemic corticosteroids for extensive disease, or severe mucosal involvement^§^   - Prednisone 0.5 – 1mg/kg/day (or equivalent dose of methylprednisolone) tapered over 2-4 weeks while instituting steroid-sparing agent | **✓** |
| Notes: Retrospective cohort studies show that in RA patients treated with TNF inhibitors there is no increase in solid cancers and no increased recurrence of breast cancer. More data are needed to determine long term lack of harm across malignancies and inhibitors.*Recommendations provided are based on case reports, series and expert consensus. Use of suggested therapies must be discussed with medical oncology based on individual patient considerations. The impact of these therapies on the anti-tumor immune response and efficacy of cancer treatment is unknown and requires further research.§ Risk/benefit must be reviewed carefully with medical oncology. | | |  |

ADL, activities of daily living; BID, twice daily; BSA, body surface area; CBC, complete blood count; CTCAE, Common Terminology Criteria for Adverse Events; HSF, Herpes simplex virus; ICU, intensive care unit; IF, immunofluorescence; IgE, immunoglobin E; PASI, psoriasis area severity index; SJS/TEN Stevens Johnson syndrome/toxic epidermal necrolysis; QID, four times daily; RA, rheumatoid arthritis; TNF, tumor necrosis factor; UVB, short wave ultraviolet B; VZV, varicella zoster virus
